# Ultra‐high dose rate radiation production and delivery systems intended for FLASH

**DOI:** 10.1002/mp.15659

**Published:** 2022-05-05

**Authors:** Jonathan Farr, Veljko Grilj, Victor Malka, Srinivasan Sudharsan, Marco Schippers

**Affiliations:** ^1^ Clinical Office Applications of Detectors and Accelerators to Medicine Meyrin Switzerland; ^2^ Institute of Radiation Physics Lausanne University Hospital Lausanne Switzerland; ^3^ Department of Physics of Complex Systems Weizmann Institute of Science Rehovot Israel; ^4^ Division of Large Research Facilities Paul Scherrer Institut Villigen Switzerland

**Keywords:** conformal, dose rate, electron, FLASH, proton, UHDR

## Abstract

Higher dose rates, a trend for radiotherapy machines, can be beneficial in shortening treatment times for radiosurgery and mitigating the effects of motion. Recently, even higher doses (e.g., 100 times greater) have become targeted because of their potential to generate the FLASH effect (FE). We refer to these physical dose rates as ultra‐high (UHDR). The complete relationship between UHDR and the FE is unknown. But UHDR systems are needed to explore the relationship further and to deliver clinical UHDR treatments, where indicated. Despite the challenging set of unknowns, the authors seek to make reasonable assumptions to probe how existing and developing technology can address the UHDR conditions needed to provide beam generation capable of producing the FE in preclinical and clinical applications. As a preface, this paper discusses the known and unknown relationships between UHDR and the FE. Based on these, different accelerator and ionizing radiation types are then discussed regarding the relevant UHDR needs. The details of UHDR beam production are discussed for existing and potential future systems such as linacs, cyclotrons, synchrotrons, synchrocyclotrons, and laser accelerators. In addition, various UHDR delivery mechanisms are discussed, along with required developments in beam diagnostics and dose control systems.

## 1 INTRODUCTION

### 1.1 High dose rates and the FLASH effect

Higher dose rates are a trend for radiotherapy machines. Higher dose rates can be beneficial in shortening irradiation times for radiotherapy and mitigating the effects of motion. The clinical flattening filter‐free linear accelerator (linac) is an example of the higher dose rate trend, delivering an average dose rate (*DR*_ave_) of about 20 Gy/min.^1^ Recently, even higher dose rates, more than 100 times greater, in the range 40–300 Gy/s (2400–6000 Gy/min), have become targeted because of their potential to generate the FLASH effect (FE).^2^ The FE is a biological effect where a differential response is observed between normal and tumor tissue when exposed to ultra‐high dose rates (UHDR). Specifically, the FE is a demonstration of reduced normal tissue toxicity and confirmation of the antitumor efficacy equivalent to that in conventional therapy dose rates.

### 1.2 Possible FE dependencies

It is important to note that UHDR and FE are related in a yet to be determined relationship. The relationship between UHDR, its application, and the observed FE are likely multifactorial and may include *inter alia*:

1. beam type
2. average dose rate (*DR*_ave_)
3. pulse dose (*D*_pulse_)
4. pulse dose rate (*DR*_pulse_)
5. beam‐on time
6. tissue type
7. tissue oxygenation
8. minimum required dose, if any
9. dose rate variations within the target volume
10. dose rate variations within the irradiated healthy tissues
11. optimal dose rate or range
12. FLASH differences in differing linear energy transfer portions of the Bragg peak for ions
13. timing matters of beam micro‐ and macrostructure
14. fractionation
15. dose conformity (or the lack thereof)
16. dose distributions overlapping in space and time
17. time interval(s) between these overlapping or nonoverlapping doses
18. relative biological effectiveness
19. FLASH differences between X‐rays, electrons, and ions
20. linear energy transfer aspects

The long list of FE unknowns presents a particular challenge for the design of a UHDR system. Despite the challenging set of unknowns, the authors seek to make reasonable assumptions to probe how existing and developing technology can address the needed UHDR conditions to provide beam generation potentially capable of producing the FE in preclinical and clinical applications. As a preface, this paper discusses the known and unknown relationships between UHDR and the FE.

### 1.3 UHDR system topics

In this paper, different accelerator and ionizing radiation types are discussed concerning the UHDR needs, which the authors expect to be relevant. The details of UHDR beam production are discussed. The discussion includes existing and potential future systems such as linacs, cyclotrons, synchrotrons, synchrocyclotrons, and laser accelerators. In addition, various UHDR delivery mechanisms are presented. The electron‐based UHDR delivery mechanisms are discussed within their specific respective system sections. Separate general proton and other ion UHDR delivery and monitoring sections are provided. Although UHDR applications may drive facility use and shielding needs, these topics also depend on local regulatory conditions and are not covered in this paper. Details on the underlying FE mechanisms and dosimetry, treatment planning, and imaging for FLASH can be found in the accompanying papers of this special issue, *FLASH: Current Status and the Transition to Clinical Use*.^3^

## 2 TERMINOLOGY

As FLASH radiotherapy is a developing field, the terminology is still evolving. The following glossary (Table 1) is provided for a consistent comparison between the concepts presented here.

**TABLE 1:** Glossary of terminology

Term	Synonym	Symbol	Definition
Average dose rate	Mean dose rate	* DR *_ave_	Dose delivered during the treatment divided by the irradiation time.
Beam			A group of particles or rays traveling in the same direction in parallel or diverging from a point.
Beam current		*I*	Unit: Ampere
Beam size			The two‐dimensional representation of a beam dimension in air or at depth in a medium. Characterized as the full width at half maximum (FWHM).
Beam macrostructure			Pulsed structure of a beam as intended for UHDR delivery, which is achieved by adding groups of smaller picosecond or nanosecond bunches.
Beam microstructure			Inherent picosecond or nanosecond bunches of radiation generated at the frequency of the accelerating RF field.
Bragg peak			The narrow, high‐dose region around the maximum dose at depths just before the end of proton or heavy ion range.
Conventional		Conv	<10 Gy/min.
Continuous beam			Beam without macrostructure pulses.
Continuous wave	Constant beam	CW	Beam output is devoid of macrostructure, but with beam microstructure in the nanosecond range providing nominally constant output over an interval of seconds or longer.
Dose modification factor		DMF	The dose modification factor (DMF) is the reciprocal of the FLASH factor. When the FLASH factor is known, the DMF might be applied to nominal dose rate doses to calculate the needed dose under flash conditions to achieve the same bioeffect under the same anatomical and physiological conditions.
Dose per pulse	Pulse dose	*D*_pulse_	The amount of dose delivered in a single macro pulse.
Double scattering	Passive Scattering	DS	In double scattering proton therapy, it is a method of spreading the beam laterally in which a pair of specially designed scattering devices consisting of a flat scatterer (first scatterer) and a contoured scatterer (second scatterer) are placed on the beam central axis. This technique has a higher efficiency of beam usage compared to the use of a single scatterer.
Energy absorber	Preabsorber, range shifter, range degrader	EA	A block of low atomic number material of uniform thickness inserted in a beam to reduce the beam's energy (and range). In some cases, an energy absorber is placed near the patient to preserve lateral penumbral sharpness.
FLASH effect		FE	Demonstration of reduced normal tissue toxicity and confirmation of the anti‐tumor efficacy equivalent to that in Conv. Implies a FLASH factor > unity. See FLASH factor.
FLASH factor	1/DMF	FF	The FLASH factor (FF) is the observed dose rate effect reduction under flash conditions. For example., if 50% reduced tissue response is observed, the FF = 2. The related dose modification factor is the reciprocal of the FF, DMF = 1/FF. Furthermore, a FLASH Factor of >1 means a FLASH effect has been observed, but it is not quantified.
Full width half maximum		FWHM	The width of a spectrum curve measured between those points on the *y*‐axis which are half the maximum amplitude.
Irradiation time		*t*	Total irradiation time in seconds.
Maximum irradiation time		*t*_max_	The maximum allowable irradiation time
Nonlinear response saturation			In ionizing radiation detection, the phenomenon that can occur when the detector/sensor no longer responds in proportion to absorbed dose, for example, due to recombination in an ionization chamber and quenching in a scintillator.
Number of pulses		*N*	The total number of pulses delivered in a single irradiation.
Particle flux	flux		The rate of transfer of particles through a unit area no. of particles/(m^2^ s).
Pencil beam scanning	Spot scanning	PBS	A technique for creating a large field by scanning a beamlet spot across the target volume. The beamlet stops at each predetermined position (“spot”) and delivers a specified dose. Irradiation is usually switched off between the points of delivery.
Plateau			The relatively uniform region of a depth‐dose distribution between the surface and the SOBP of a range‐modulated beam or between the surface and the Bragg peak of a nonrange‐modulated (pristine monoenergetic) beam.
Pulse dose rate	Instantaneous dose rate	* DR *_pulse_	The dose rate achieved in a single pulse.
Pulse repetition frequency	Pulse repetition rate	PRF	The number of pulses (*N*) delivered over a designated period of time.
Radiofrequency	RF	*f*_RF_	A frequency in the range 10^4^ to 10^11^ or 10^12^ Hz.
Range degrader	Range shifter	RD	The same function as an energy absorber (EA), but range degraders are associated with a permanent installation within the system and EAs are “add‐ons” to the system, that is, RDs may not be removable from the system, but EAs are.
Range modulator			A range modulator that consists of several ridges and valleys that present different thicknesses of material to an incoming beam to vary its penetration into the patient.
Ripple filter	Ridge filter		A range modulator (typically a thin ridge filter) that produces just enough variation in the light ion energies entering the patient that a reduced number of accelerator energies may be used without producing ripples in the depth‐dose distribution.
Shoot‐through	Transmission	ST	An irradiation technique for ions where the beam penetrates through the subject, stopping behind, that is, outside the subject, using the plateau region of the Bragg curve for dose deposition within the subject.
Ultra‐high dose rate		UHDR	Average dose rate >40 Gy/s across macro pulses, not DR_pulse_.

## 3 CRITICAL FLASH PARAMETERS AND SYSTEM REQUIREMENTS

### 3.1 Introduction to critical FLASH physical parameters

Despite an immense interest in FLASH radiotherapy (FLASH‐RT), sparked by Favaudon et al. in 2014,^2^ the precise beam characteristics required to reproduce the FE at increased dose rates are still far from being understood. There are two main reasons for this. One lies in the fact that regardless of numerous literature reports attributing the name FLASH to different machines, the number of radiation beams validated to reproduce the FE remains very low. As strongly recommended by Vozenin et al.,^4^ the terminology “FLASH beam” or “FLASH irradiator” should be adopted only when the FE has been shown in vivo. This includes demonstrating reduced radiation toxicity in healthy tissues and equivalent or better anti‐tumor efficacy than conventional (Conv) treatment. The second reason is that almost all published studies that have investigated the benefits of FLASH‐RT compared tissue responses between the Conv mode and a small number (often only one) of UHDR modes. Detailed research of the FE as a function of gradually changing temporal beam structure is highly time consuming and requires numerous animals, which also entails the necessary ethical approvals. The situation is complicated further by the large diversity of tissues (skin,^5^, ^6^ brain,^7^ lung,^2^ intestine^8^) included in preclinical studies and the potential tissue‐specific dependency of the FE on beam properties.

#### 3.1.1 Temporal structure of FLASH beams

A unique characteristic of FLASH beams is reflected in their temporal structure, which can be described at the level of micro‐ and macrostructure. The microstructure is an inherent property of all beams (electron and proton/ion) accelerated with radiofrequency (RF) electromagnetic fields. It consists of picosecond or nanosecond bunches of radiation generated at the frequency of the accelerating RF field. The importance of beam microstructure for the occurrence of the FE has not yet been investigated. On the other hand, macrostructure refers to the pulsed nature of the (usually electron) beam, which is achieved by arranging smaller bunches into microsecond pulses. If the macropulses are absent, the beam is classified as (quasi)continuous. The average dose rate commonly describes the rate at which the dose was delivered to the target. While being well suited for characterizing continuous (proton) FLASH beams, the use of average dose rate becomes questionable in the case of pulsed FLASH beams composed of a small number of microsecond pulses (*N *< 10), spaced tens of milliseconds apart. Instead, such beams are better characterized by the total irradiation time and dose per pulse (typically above 1 Gy). Critical parameters for obtaining the FE with electron and proton beams will be considered separately due to their significantly different temporal structure (pulsed vs. continuous).

#### 3.1.2 Critical parameters for electron FLASH beams

**FIGURE 1:**
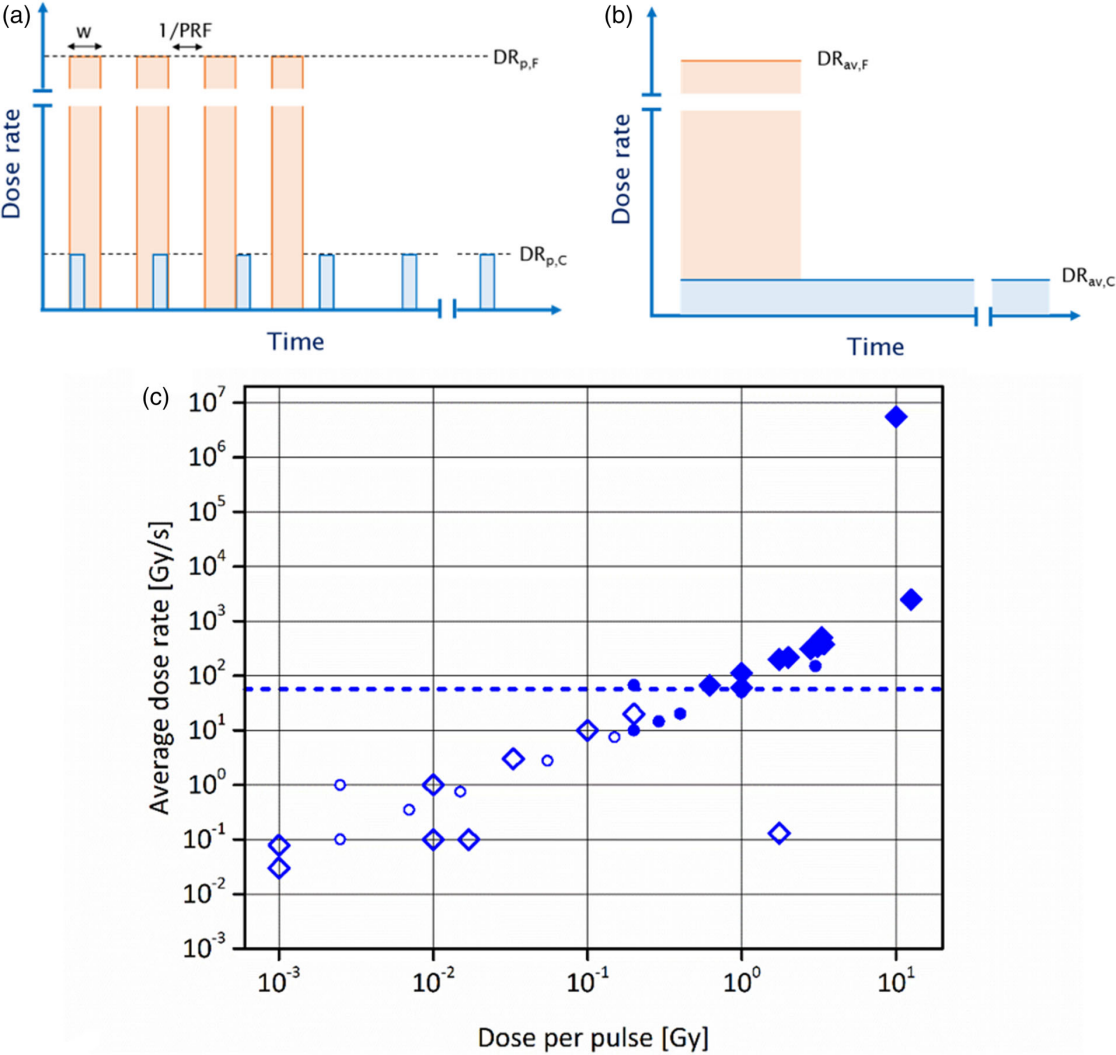
Schematic illustration of the beam structure of (a) pulsed and (b) continuous radiation beams. PRF, pulse repetition frequency; w, pulse width; *DR*_p,F_, pulse dose rate of FLASH beam; *DR*_p,C_, pulse dose rate of conventional beam (beam type specific instances of the general *DR*_pulse_); *DR*_av,F_, average dose rate of FLASH beam; and *DR*_av,C_, average dose rate of conventional beam (beam type specific instances of the general *DR*_ave_). Both, pulsed and continuous beams, possess inherent microstructure composed of the nanosecond bunches generated at the frequency of the RF field (not shown in the illustration). (c) Observed characteristics leading to sparing of the normal tissue with UHDR electron and proton beams. The filled markers represent combinations of *DR*_ave_ and *D*_pulse_ that lead to reduced radiation toxicity of pulsed electron beams in healthy tissues in comparison with conventional low dose rate radiation. The hollow markers indicate at which combinations of the same parameters no reduction in toxicity was observed. Points that belong to FLASH‐validated electron beams (with proven antitumor isoefficacy^2,^ ^5^, ^6^, ^7^, ^8^, ^13^, ^15^) are depicted with larger diamond markers, whereas other electron beams (tumor response not investigated^11^, ^12^) are represented with smaller circles. The dashed blue line indicates the lowest *DR*_ave_ at which the FLASH effect was reported with proton beams

Preclinical research on FLASH‐RT was predominantly conducted with energetic (4.5–20 MeV) pulsed electron beams, grouped in pulses of 0.5–4 µs duration at a rate of 5–200 pulses per second.^2^, ^5^, ^6^, ^7^, ^8^, ^9^ When operated in FLASH mode, such beams deliver treatment doses in a fraction of a second by a small number of pulses. In contrast, delivery of the same doses with Conv beams requires irradiation times measured in minutes. Irradiation time is inversely proportional to the mean *DR* (*DR*_ave_), which is a frequently used parameter to describe the rate by which the dose is deposited to the target. However, the concept of the *DR*_ave_ is questionable for pulsed electron FLASH beams, due to the small number of pulses (usually *N* < 10) that carry relatively high doses each. The time structure of the beam then resembles timely fractionated dose administration, where several instantaneous fractions of one or more Gy are spaced ten or more milliseconds apart. A large dose per pulse (*D*_pulse_ > 1 Gy) defines another property specific to pulsed electron FLASH beams. The situation is opposite in Conv mode with *D*_pulse_ smaller than 0.01 Gy, at least two orders of magnitude lower than in FLASH mode. Consequently, several hundred or even thousands of pulses are required to deliver the total dose in Conv mode. A striking difference in total irradiation time and *D*_pulse_ between Conv and FLASH modes implies the crucial role of those parameters in evoking the FE with electron beams. In the only reported systematic dose rate escalation study that was performed with the use of validated FLASH beam, it was demonstrated that the sparing of healthy mouse brain was entirely lost when the irradiation time was prolonged over 0.5 s (*DR*_ave_ < 20 Gy/s) and *D*_pulse_ lowered below 0.2 Gy.^7^ Unfortunately, it is not possible to conclude from this study which of the two parameters (if any) is more important for the observed biological effect because, during the de‐escalation of the beam from FLASH to Conv mode, both parameters were altered simultaneously. Remarkably, the thresholds for beam ON time and dose per pulse, established by Montay‐Gruel et al.,^7^ apply to other electron beams shown to elicit healthy tissue sparing at UHDR.^2^, ^8^, ^10^, ^11^, ^12^, ^13^ Except for one study from 1982,^14^ which used a very high dose of 65 Gy for irradiation of mouse tails, the reduction in electron beam efficacy against healthy tissues was never observed with exposures lasting longer than 0.4 s, or with *D*_pulse_ lower than 0.2 Gy. Figure 1, based on electron and proton preclinical FLASH investigation results, compares the data from electron investigations demonstrating the FE with tumoricidal isoefficacy,^2^, ^5^, ^7^, ^8^, ^9^, ^10^, ^15^, ^16^ and those where, although the FE was observed for normal tissues, tumoricidal effects were not studied.^12^, ^14^ Figure 1 attempts to correlate *DR*_ave_ with *D*_pulse_ for electron beams. In addition, there exists an inherent microstructure defined by the RF, which exists for both pulsed and continuous beams. This microstructure consists typically of nanosecond bunches, which are not indicated on the drawing and are not currently considered regarding defining the critical properties of FLASH beams. Also, the lowest reported proton *D*_ave_ producing the FE is included as an additional reference.

#### 3.1.3 Critical parameters for proton FLASH beams

In addition to pulsed electron beams, the FE was confirmed with cyclotron‐generated proton beams having energies between 230 and 250 MeV.^17^, ^18^, ^19^ Concerning the time structure, these beams can be classified as quasi‐continuous beams consisting of nanosecond bunches generated at frequencies of around 100 MHz. In the absence of a more focused approach on beam microstructure, the average dose rate/irradiation time represents the main critical parameter distinguishing FLASH proton beams from Conv proton beams. Diffenderfer et al.^17^ registered the FE with a *DR*_ave_ of about 80 Gy/s. That value is compatible with the report by Zhang et al.,^20^ who used 120 Gy/s to achieve reduced toxicity to the intestine of mice. Especially interesting is that the two mentioned studies employed passive scattering to cover the target uniformly with the beam. The FE was more recently confirmed in a pencil beam scanning (PBS) configuration.^19^ The authors reported the effect at the *DR*_ave_ of 57 and 115 Gy/s, computed as the ratio between the total dose (35 Gy) and total irradiation time. Still, the tissue's actual dose rate was several times, if not even an order of magnitude, higher. The situation with PBS is complicated by the spatial overlap of doses from neighboring spots. As a result, the dose rate inside the target is not homogeneous and depends on contributions from different spots. There have been several attempts^21^, ^22^ to define the proper metric for quantifying spatially varying dose rate distribution of FLASH PBS treatment plans. The theory continues to develop; a consensus model has not been reached. Nevertheless, the number of spots contributing to any small tissue volume (voxel) is undoubtedly lower than the total number of spots used, which means that each voxel is irradiated for a time duration shorter than the total irradiation time. Thus, although variable, dose rate in the voxel is always higher than the average dose rate. Also, the pulse dose *D*_pulse_ and pulse dose rate *DR*_pulse_ should be considered for pulsed ion accelerators.

#### 3.1.4 Critical parameters for X‐ray FLASH beams

Achieving UHDR X‐rays with standard clinical linacs is exceptionally challenging due to high losses in the conversion target. Previously, a synchrotron light source (SLS) demonstrated normal mouse brain sparing at a *DR*_ave_ of 37 Gy/s.^7^ Because the irradiation was performed using a narrow slit that collimated the X‐ray beam in a slice of 50 µm width, the actual tissue's *DR* (*DR* in the slice) was around 12 000 Gy/s. Currently, there are no data that would indicate the minimal dose rate for X‐ray FLASH beams.

### 3.2 Summary of critical FLASH physical parameters

For electron beams, the data on critical FLASH parameters are genuinely scarce, with only a few beams having reproduced the FE in vivo. The trends observed from the literature on pulsed electron FLASH beams indicate the irradiation time and the *D*_pulse_ as two beam properties that are critical for reducing the radiation toxicity in normal tissues. Irradiation times shorter than 0.4 s and *D*_pulse_ higher than 0.2 Gy are required to achieve the FE with electron linacs. The individual contribution of these two properties to the biological response is yet to be resolved.

Regarding FLASH parameters for proton beams, due to the quasi‐continuous temporal structure of cyclotron proton beams, the *DR*_ave_ is a significant critical beam parameter for proton FLASH‐RT. There may also exist a dose threshold (delivered at UHDR), below which the FE is not observed. Dose values higher than 80 Gy/s seem to be enough for eliciting the FE in double scattering (DS) beam irradiations. The lowest dose rate below which the effect diminishes is unknown.

With PBS dose delivery, parameters such as *DR*_pulse_ are also expected to be relevant, where *DR*_ave_ drops quickly due to the overall scanned irradiation time, whereas individual spots may still be delivered at UHDR. Here, we retain the general concept of *DR*_pulse_ for proton beam “spot” deliveries that may consist of one pulse per spot, or multiple pulses per spot for pulsed systems. Indeed, the roles of scanning speed and spot size on the FE with PBS remain to be resolved, and the critical FLASH parameters are still evolving for ion PBS systems.

The only study that demonstrated the sparing effect of X‐rays at UHDR was conducted at the European Synchrotron Radiation Facility (ESRF), Grenoble, France, with a specific spatial beam configuration that included a narrow, 50 µm wide slice of the high‐intensity beam. The *DR*_ave_ in the slice was around 12 000 Gy/s. Like the situation with proton beams, no *DR* de‐escalation study was performed to determine the threshold DR for X‐ray FLASH‐RT.

### 3.3 Preclinical FLASH system requirements

Preclinical irradiation systems are highly customizable experimental platforms optimized for irradiations of small animals, usually rodents, and various in vitro samples like cells or artificially grown tissue models. Small irradiation fields, simple beam delivery, easy adaptation for irradiation of different samples, tunable beam parameters, and lower costs and safety requirements than clinical machines typically characterize this type of radiation device. The required beam energies are substantially lower than clinical therapeutic beams, and radiation field sizes of a few centimeters are usually sufficient for covering the targets of interest. These conditions are shared among all preclinical irradiators, regardless of dose rate considerations. However, what distinguishes FLASH machines is the need for high beam intensities to reach the UHDR range and deliver the treatment dose in a fraction of a second. Because the minimal requirements for eliciting the FE are unknown, due to the lack of systematic parametrization studies, any preclinical FLASH irradiator must aim to generate the beam with a time structure like the temporal characteristics close to those of an already validated FLASH beam. In addition, it is favorable for such machines to support the gradual escalation of the average dose rate and, in the case of electron beams, the dose per pulse, from conventional mode up to UHDR. This feature is crucial for studying the dependence of the FE on different beam parameters. The exact temporal characteristics that should be met in a preclinical FLASH setting directly follow from the considerations presented in Section 3.1 and strongly depend on the type of radiation.

In the case of electron beams, the temporal macrostructure of the beam must be considered. Preclinical electron irradiators should be capable of delivering pulsed beams with parameters described in Table 2. Doses of more than 1 Gy contained in a microsecond pulse have so far only been achieved with several experimental accelerators. They have also been shown to be accessible by standard clinical linacs after certain modifications.^23^, ^24^ Furthermore, pulsed electron UHDR beams pose substantial challenges for dosimetric procedures, due to saturation experienced by the standard transmission ionization chambers (TICs). Fortunately, the preclinical setting does not necessitate the use of online monitor chambers and allows for the determination of the dose with passive dosimeters (films, alanine pellets, and TLDs). It is worth noting that, in the preferred case, the UHDR regime would be accessible for electron beams solely by ramping up the pulse repetition frequency (PRF), while keeping the *D*_pulse_ unchanged. This would allow for decoupling and independent evaluation of the two‐beam properties, the irradiation time and *D*_pulse_, which proved to play a vital role in reproducing the FE. However, due to the limitations of current electron linacs, it is impossible to reach the UHDR mode with pulse doses typical of the Conv mode (<0.01 Gy).

**TABLE 2:** Critical preclinical FLASH parameters

	Electron	Proton
Dose per macro pulse, *D*_pulse_	>1 Gy	>4 Gy
Pulse duration	0.5 < *w* < 4 µs	<100 ms if applicable
Maximum irradiation time, *t*_max_	0.4 s	0.3 s
PRF	>10 Hz	NA
* DR *_ave_	>100 Gy/s	>40 Gy/s*
*D*_min_	8 Gy	Not yet established
Beam energy	>4.5 MeV	>35 MeV for SOBP
		>70 MeV for transmission
Beam size (95% isodose)	>2 cm	>2 cm for passive scattering
		min/max spot size not yet established for PBS systems

* *Note*: Although the minimum proton DR_ave_ 40 Gy/s is frequently referenced in the literature, the authors are not aware of any induced proton FE results obtained at less than 60 Gy/s. And the “minimum” proton DR_ave_ inducing the FE remains to be determined. For consistent comparison with other publications, a minimum DR_ave_ of 40 Gy/s is also used here.

For DS, the requirements for preclinical proton beams (Table 2) are mainly driven by the *DR*_ave_, which should surpass 40 Gy/s to be compatible with proton dose rates that have already reproduced the FE. Standard, cyclotron‐based, proton facilities were repeatedly confirmed to achieve such dose rates over field sizes compatible with preclinical targets, either in DS or PBS configurations.^17^, ^18^, ^19^ The pulse duration is reflective of established values from PBS systems. The relationships between the parameters are still under investigation. The minimal required proton energy depends on the dose delivery method. If a shoot‐through method is used, the energy must be high enough (>70 MeV) for the Bragg peak to fall outside the animal. Much lower energies (>35 MeV) are needed in the case when the dose is delivered by the spread‐out‐Bragg‐peak (SOBP).

### 3.4 Clinical FLASH system requirements

The base requirements for clinical FLASH systems, in terms of the temporal beam characteristics, are shared with preclinical devices and defined by UHDR conditions that reproduce the FE. However, the size of the radiation field and beam energy must be scaled up according to the size of clinically relevant targets. Shallow penetrating electron beams with energies between 4 and 10 MeV, that were predominantly used in preclinical FLASH studies, are limited only to superficial and intraoperative treatments in a clinical setting. With very high energy electron (VHEE; >100 MeV) and X‐ray UHDR beams still being in the early developmental phase, protons are the only FLASH‐validated radiation modality available immediately to treat deep‐seated tumors in the clinic. This paper explores how these requirements might be realized with modifications to existing and new systems. Additional requirements for clinical application of FLASH‐RT include clinical beam delivery (Sections 8 and 9), image guidance for precise dose delivery (“*Ultra‐High Dose Rate Dosimetry: Challenges and Opportunities for FLASH Radiation Therapy*”, in this special issue), and online dose monitoring (Section 10) coupled with appropriate safety protocols (“*A Roadmap to Clinical Trials for FLASH*”, in this special issue).

## 4 UHDR ACCELERATORS

UHDR systems include those capable of producing X‐ray, electron, proton, and potentially heavier ion beams. Preclinical investigations have used X‐rays, electrons, and protons. Protons^25^ and electrons^26^ were the first to be used to treat patients with FLASH‐RT. Cyclotrons, synchrotrons, and linacs are used to produce UHDR beams; further UHDR‐based development is ongoing for each accelerator type, and laser‐based systems are also being developed. The following sections describe the underlying technologies and developments for accelerators, and compare the potential advantages and disadvantages, for UHDR beam production by the different circular (Section 5), linear (Section 6), and laser (Section 7) accelerators.

## 5 CIRCULAR UHDR ION ACCELERATORS

In radiation therapy, the advantages of protons and heavier ions are the combinations of a limited range, the highest dose deposition just in front of their range, and (depending on depth and ion) limited lateral scattering. Dose distributions have been improved by exploiting these properties of proton and ion beams. In general, in particle therapy, the dose to healthy tissue is considerably reduced compared with the most widely applied radiation therapy with high‐energy X‐rays.

The current particle accelerators’ applications will be discussed for their possible use in FLASH irradiation therapy with protons or carbon ions. At present, in most facilities, the proton or ion acceleration systems used are the synchrotron, or one of the two versions of the cyclotron: the isochronous cyclotron or the synchrocyclotron. According to the current Particle Therapy Co‐Operative Group statistics (https://www.ptcog.ch/index.php/facilities‐in‐operation), there are 58 cyclotrons, 13 synchrocyclotrons, and 37 synchrotrons currently in operation worldwide. The significant differences between these three accelerator types are the footprint of the accelerator, how and at what speed the energy of the beam is varied, and the time structure of the beams and the beam intensity. Here, the FLASH‐therapy‐related concepts of the three types of accelerators and the FLASH‐relevant properties of their accelerated beams will be discussed.

In this section, some key numbers are used to enable reasonable comparisons between the capabilities of the different accelerators. Furthermore, it is also assumed that the typically needed proton dose rate to obtain a FE is approximately 40 Gy/s and that it should be given within a so‐called FLASH pulse of <100 ms. In this section, two cases of FLASH irradiations are considered, representing the currently expected extremes: the so‐called preclinical and the clinical cases. In the case of experiments, a volume of 1 cm^3^ is irradiated under FLASH conditions to give a dose of 2 Gy. The other case is more related to clinical situations, in which a target of 1 kg should receive a dose of 2 Gy under FLASH conditions. Although there are indications that a minimum dose of 3–10 Gy is required to get the advantages of FLASH,^27^, ^28^ we will simply assume a dose of 2 Gy is to be given to obtain the order of magnitude of relevant parameters and for comparison.

For these two FLASH applications, the two cyclotron types and the synchrotron will be compared. Because it will be made clear how these comparisons are made, it will be easy for the reader to scale the outcomes to another dose, volume, dose rate, and so on.

### 5.1 The proton cyclotron, synchrocyclotron, and synchrotron for UHDR irradiations

In proton therapy facilities, synchrotrons and cyclotrons accelerate protons up to energies in the 200–250 MeV range, and carbon ions are accelerated in synchrotrons up to 430 MeV/nuclide. These energies are needed to achieve the clinically relevant ranges of 25–30 cm in tissue. The time structures and the intensities of the beam are quite different for the three accelerator types. The isochronous cyclotron delivers a beam that can be regarded as continuous. Still, synchrocyclotrons produce pulsed beams at a PRF in the range of 500–1000 Hz, whereas beams from synchrotrons are extracted within several seconds, as a more or less continuous beam. This so‐called spill from the synchrotron is repeated after approximately a few seconds. These are essential beam properties to be discussed here since these may play a vital role in achieving the FLASH conditions.

After a basic description of each accelerator, we will discuss the FLASH relevant properties of cyclotrons and synchrotrons: the time structure of the beam, beam intensity, and variation of beam energy. More details on the different accelerators can be found elsewhere.^29^, ^30^ It is convenient for those not in the accelerator field to know that a proton beam intensity is expressed as an electrical current, so in nanoamperes (nA), microamperes (µA), or milliamperes (mA), and the number of protons as a charge in nanocoulombs (nC).

The accelerators discussed here are circular accelerators, in which the particle beam is repeatedly crossing gaps with strong electric fields in which the acceleration takes place. Due to the high speed of the particles, the electric fields (RF fields) oscillate at a high (radio) frequency (tens of MHz). The particles must cross in these acceleration gaps many times to obtain high energy. A magnetic field is used to guide the particles along a circular orbit, crossing the acceleration gaps so that the particles will cross them repeatedly. When the particles are crossing the gaps, it is essential that this is just at the moment when the electric field strength in the gap is at its appropriate value (i.e., at the corresponding right RF‐phase), so that the particles are accelerated when crossing the gaps.

### 5.2 Basic concept of a cyclotron

Cyclotrons used in particle therapy accelerate protons. Although cyclotrons for heavier ions (e.g., carbon ions) are in development, we will only discuss proton cyclotrons.

A cyclotron consists of a large cylindrical volume with a strong and almost homogeneous magnetic field parallel to its axis, guiding the protons in nearly circular orbits around the axis. Some (1, 2, 3, or 4) Dee‐shaped electrodes are mounted at a voltage in the cylindrical volume, oscillating at the RF. These will increase the proton energy when they cross the Dees at the proper RF‐phase oscillating voltage. The orbit radius of the protons will increase with the energy of the protons. The protons are created in an ion source in the center of the machine. Coming out of the ion source, they are bent in the magnetic field. Due to the acceleration, they follow a spiral track over several hundred (in isochronous cyclotrons) or several thousand (in synchrocyclotrons) turns, until they are extracted from the cyclotron at the right energy. The strength of the magnetic field determines the energy of the extracted proton beam and the achieved orbit radius at which the protons are extracted. In the currently existing machines for proton therapy, these values cannot be varied. Therefore, the energy of the protons is machine specific and, for example, 200, 230, or 250 MeV.

The essential operation principle of the isochronous cyclotron is that the time *T* for a proton to make a complete circle (= one turn in the cyclotron) is equal for all proton energies (*isochronous acceleration*). For protons with charge *q* and mass *m*, in a cyclotron operating at magnetic field *B*, the orbit revolution time is:

(1) $$T=\frac{2\pi m}{B\cdot q}$$

The acceleration gaps (the Dee‐edges) are oriented along the radial direction, perpendicular to the proton motion. At the appropriate phase concerning the RF field, all protons must cross a gap at that moment. Therefore, in the cyclotron, all protons must always be at approximately the same azimuth (= angular position) to get approximately the same energy gain per gap crossing, but at different radial positions, which depend on their energy. The RF must also be equal to an integer multiple (*h*, the harmonic number) of the orbit revolution frequency 1/*T* to have the protons always crossing the acceleration gaps at the proper phase of the RF field.

(2) $$\mathrm{RF}=h\frac{1}{T}=h\frac{B\cdot q}{2\pi m}$$

Typically used RF is in the range of 10–100 MHz. Protons extracted from the cyclotron will be grouped (bunched) at this RF. However, since the rate of these bunches is very high (a bunch at every 1/RF = 100–10 ns), they cannot be distinguished in most processes. Therefore, this continuous wave (CW) of protons can usually be approximated as a continuous beam. Because the ion collection time (100 µs) in ionization chambers is order of magnitude larger than the pulse duration for cyclotrons (ns), the beam can be considered as continuous for saturation effects.^31^

#### 5.2.1 Isochronous cyclotron

Originally, cyclotrons have been designed with a homogeneous magnetic field *B*. However, at energies above approximately 30 MeV, one must take the relativistic effect of mass increase into account: *m* = *γ·m*_0_, with *m*_0_ = rest mass of the proton. Following Equation (1), the relativistic factor, *γ*, is unity at low energy, but increases to *γ* = 1.27 for 250 MeV protons. This increase of *m* would reduce the orbital revolution frequency 1/*T* by 21% at this energy. In the commercial isochronous cyclotrons, this is corrected by an increase of *B* with radius (i.e., energy): *B*(*r*) = *γ*(*r*), *B*(*r* = 0).^32^ With this correction of the magnetic field, the proton revolution time *T* in Equation (1) remains constant (isochronous) with increasing energy and stays in phase with the RF field. The continuously extracted beam has the CW character discussed before, with 500–1000 nA typical maximum intensities.

#### 5.2.2 Synchrocyclotron

In a synchrocyclotron, the magnetic field is usually homogeneous or decreases a bit with radius. This is when powerful superconducting (SC) magnets are used, like in the very small SC synchrocyclotrons developed for proton therapy.^33^ From Equation (1), it follows that the combination of *B* decreasing with *r* and the relativistic increase of *m* will decrease the revolution frequency 1/*T* of the protons with increasing energy (i.e., radius).

The RF is varied in time to keep it matching (staying synchronous) to the decreasing revolution frequency of a group of accelerated protons, per Equation (2), to compensate for this decreasing revolution frequency during acceleration. This decreasing RF is matched to the revolution frequency of this group only, so that it will accelerate this group from cyclotron center to extraction. During this acceleration time interval, the RF will be too high or too low for protons at higher or lower radius (or energy), respectively, which will not be accelerated. After reducing RF and extraction of the matching group of protons, the RF increases and sets back to the starting value, matching the protons in the cyclotron center again. This process of RF modulation is repeated at a rate of a few hundred up to a thousand Hertz, depending on the cyclotron. The group of protons being accelerated covers the range of radii, matching the RF. Therefore, during the few microseconds in which these protons are extracted from the cyclotron (which we will call RF mod‐pulse here), the beam intensity has a similar bunch structure at RF as the beam from the isochronous cyclotron.

Because only during <1% of the time (i.e., every 1–2 ms a pulse of a few µs), the beam is coming out of the synchrocyclotron, one needs an extracted beam intensity of approximately a µA during the RF mod‐pulse (i.e., 0.01 nC/pulse), to extract an average beam intensity in the order of 10 nA.^34^ As shown below, the needed dose rate with FLASH irradiations can thus be achieved in experiments on small targets. Still, the required FLASH conditions cannot be fulfilled in typical clinical irradiations. It is unknown if the time interval of 1–2 ms between the pulses plays a role in the processes causing the FE.

### 5.3 Basic concept of a synchrotron

Synchrotrons and cyclotrons are commonly used for proton therapy, but synchrotrons are the only machines used currently for heavy ion therapy. Typical for a synchrotron is that the particles are accelerated until the desired energy has been reached, and the intensity of the extracted beam is independent of its energy.

The beam is created in a separate ion source, preaccelerated in a linac, and injected into a ring. The bending magnets and focusing magnets guide the particles’ bunches along a closed, approximately circular orbit in the ring. The ring can be filled up to its (ring‐dependent) maximum capacity of approximately 10^9^–10^12^ particles. When the ring has been filled, injection is stopped, and acceleration is started. Acceleration is performed by one or more RF cavities (RF is several MHz) in the ring. These are switched on until the desired energy of the particles is reached. The particle energy (the momentum *p*) is linked to the ring's bending (and focusing) magnetic field strengths. The magnetic field strength *B* of the magnets is increased synchronously to the increase of the particle momentum *p* in such a way to keep the particles with charge *q* in their orbit with radius *r* during the acceleration process according to:

(3) $$\frac{p}{Bq}=r=\mathrm{const}.$$

When the desired energy has been reached, acceleration and increase of the magnetic fields are stopped, and the beam is stored in the ring. Then the so‐called slow extraction process is started, in which the extracted beam is “scraped off” from the beam stored in the ring. Depending on the ring filling and the required dose rate at the patient, this can take between a few tenths of a second up to several seconds. Then, the magnets in the ring are ramped down, and the whole process (often called a spill) is repeated at the same or another energy.

During the filling of the ring, the acceleration, and ramping down processes, no beam is extracted, so the beam intensity at the patient will be zero during these moments.

The regular slow (“multiturn”) extraction process takes 0.5–5 s. The beam intensity from a synchrotron is subject to intensity fluctuations of up to 50%. The fluctuations of several kHz, up to a few MHz, follow the filling pattern, the RF, and beam orbit oscillations in the ring. Several groups are working on improvements of the beam intensity stability.^35^, ^36^ Depending on how fast the beam is extracted from the ring, the extracted beam intensity is usually between 0.1 and a few nA.

Extraction from a synchrotron can also be done in single turn, in which all particles are “kicked out” of the synchrotron ring in one turn, so within a fraction of a microsecond. This gives a beam intensity in the order of 100–1000 µA during the extraction. However, in single extraction mode, one or more seconds are needed until the next pulse can be extracted.

### 5.4 Beam intensity and time aspects in UHDR irradiations

**FIGURE 2:**
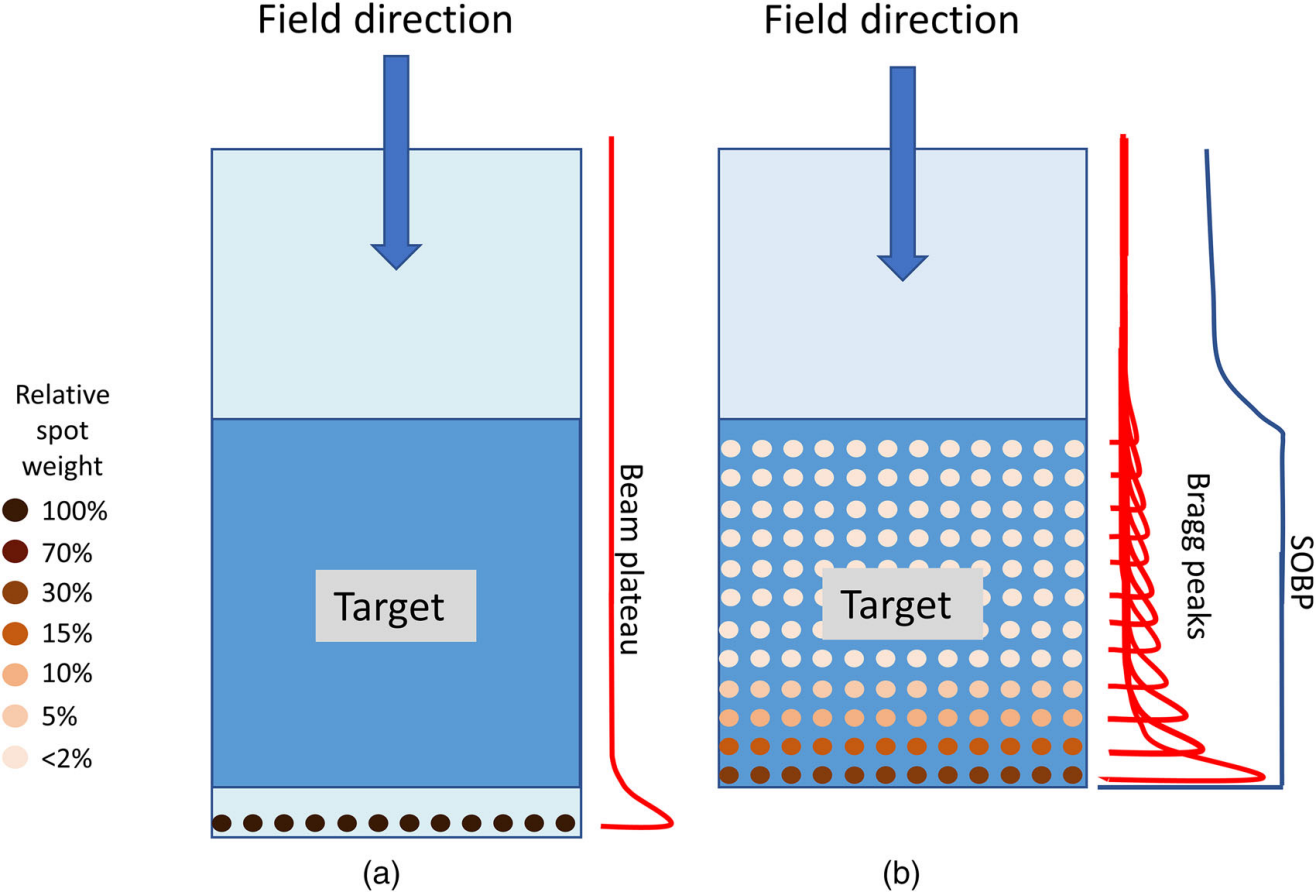
Transmission fields using single energy are depicted for a 1 cc and 1 L field (a), and the related SOBP fields are shown (b). The transmission field protons stop beyond the target, and the SOBP field protons stop in the target using multiple energies. Even the 1 cc SOBP field requires more than one energy. Although the SOBP fields in this figure are depicted using PBS, SOBP fields can also be formed by combinations of scatterers, beam scanning range modulators, and ridge filters (see Section 9)

An example is presented, based on typical conditions to perform FLASH experiments, to illustrate how the beam intensities from the different accelerators match the FLASH requirements. This is followed by an example based on clinically used typical requirements. In both examples, crude approximations are used to obtain estimates of the relevant quantities. Again, although a minimum dose may exist to observe the FE, 2 Gy is chosen for simple calculation, as a readily recognized clinical dose that can be scaled by the reader for higher dose timing comparisons. The example irradiation fields are presented in Figure 2.

The different accelerators will be evaluated based on the conditions used in these examples, which are discussed below and listed in Tables 2 and 3.

1. In the considered FLASH experiment, we assume that a water equivalent (WE) target of 1 cm^3^ (in a small animal) tissue is irradiated with the shoot‐through technique (Figure 2(a)). The so‐called plateau dose is deposited in the target instead of the high Bragg peak dose. Here, we assume that a proton beam of, for example, 230 MeV is used in this shoot‐through technique. In that case, the average energy loss of each proton will be approximately 4 MeV at the target. To deposit a dose of 2 Gy in 1 cm^3^ (= energy deposition of 0.002 J), approximately 3 × 10^9^ protons or 0.5 nC are needed. The required FLASH dose rate of 40 Gy/s is achieved if the 2 Gy (i.e., the 0.5 nC) are given in a FLASH pulse of <50 ms. During the FLASH pulse of 50 ms, this would need an average proton beam intensity of approximately 10 nA at the target, spread over 1 cm^2^.
2. The following, more clinically oriented, example is based on typical values currently used in a clinic: 2 Gy in a target of 1 kg (i.e., an energy of 2 J is deposited in the target). For a homogeneous SOBP dose distribution in a 1 kg WE tissue target, energy‐modulated protons from a ∼230 MeV beam are stopped in the target (Figure 2(b)). In the target volume, the average energy deposition per proton can be approximated to be 60 MeV, so that for 2 J energy deposition, approximately 2 × 10^11^ protons or 30 nC are needed. If the 2 Gy (i.e., the 30 nC) are given within 50 ms, the required dose rate of 40 Gy/s is achieved by an average beam intensity of approximately 600 nA at the patient. It is important to realize that this intensity is averaged over all modulation energies. Furthermore, this average intensity during the irradiation FLASH pulse of 50 ms is approximately a factor 1200 higher than the 0.5 nA currently needed to administer 2 Gy/kg/min to a patient. In addition, the aim is to induce the FE in the healthy tissue, not necessarily in the target. In this example, the healthy tissue is in the region proximal to the SOBP. As such, in this example, the required beam intensity to achieve the FE in healthy tissue may be underestimated by the ratio of the SOBP to entrance plateau. Also, it will be assumed that the 2 Gy are delivered homogeneously over the target volume, and no attempt has been made to describe how the dose rate is distributed over the target. In a recent paper by Folkerts et al.,^37^ a method is proposed to calculate the dose rate distribution of a pencil beam proton field.

**TABLE 3:** Conditions of FLASH irradiations, used for comparing accelerators

(A) FLASH conditions for experiments	
Dose	2 Gy
Target volume	1 cm^3^ water equivalent
Beam energy at the target	230 MeV
Average energy loss per proton in target	4 MeV
Needed average beam intensity at the target	10 nA
Equivalent charge per FLASH pulse	0.5 nC
(B) FLASH conditions for clinical irradiation	
Dose	2 Gy
Target volume	1 L water equivalent
Beam energy at the target	70–230 MeV (modulated)
Average energy loss per proton in target	60 MeV
Needed average beam intensity at the target	600 nA
Equivalent charge per FLASH pulse	30 nC

Using the transmission and SOBP fields as base concepts, the following will be discussed: if and how the circular accelerators can achieve these FLASH‐related intensity requirements for experiments and clinical irradiations in principle. This will yield a fundamental starting point in the technological developments for FLASH irradiations. In addition, apart from the accelerator, aspects such as range modulation, PBS, and gantry angle will have to be considered and adapted.

### 5.5 Applicability of isochronous cyclotrons for UHDR irradiations

Most cyclotrons in proton therapy use an internal proton source mounted at the center of the cyclotron. Here, a small volume is filled with hydrogen gas. Ionizations create plasma in an arc current (a continuous “sparking”) in the gas volume. A tiny opening in the wall of the gas enclosure allows protons to leave the plasma. Beam intensity regulation can be performed by adjusting the arc current and the gas flow in the source. However, the reproducibility and reaction speed are limited. Therefore, in several cyclotrons, a method is used to partially stop the beam at a collimator in the central region. The needed beam deflection is achieved by either an electrostatic deflector or by a variation of the RF voltage.

In principle, relatively high beam intensities (several mA) can be achieved directly from ion sources. However, only a fraction of this can take part in the acceleration in a cyclotron, due to the limited phase width accepted by the RF. The typical maximum CW beam intensities extracted from medical isochronous cyclotrons are 500–1000 nA. Higher intensities can be achieved by increasing the output from the ion source and, at the same time, by reducing beam losses during acceleration and extraction. These losses cause much of the intensity limitations, and many cyclotron development groups/companies are working on reducing these losses to enable higher intensities in the future.^38^

When using an isochronous cyclotron, FLASH experiments are possible at the beam energy extracted from the cyclotron. With, for example, an intensity of 500 nA at the 1 cm^3^ target, the required 0.5 nC can be sent through the target within 1 ms. This yields a dose rate of 2000 Gy/s. If necessary, one can benefit from the allowed maximum irradiation time of 100 ms to compensate for the eventual 100 times lower beam intensity one gets due to transmission losses when using lower proton energies by degrading the beam from the cyclotron.

For the example of clinical use, the required 30 nC for the 2 Gy dose in a target of 1 kg can be achieved with a *DR*_ave_ of 40 Gy/s, if applied within 50 ms. This requires an average beam intensity of 600 nA at the patient. However, to achieve this average intensity at the patient, the time to change energy and eventual beam transmission losses due to the energy modulation must be considered. These can only be compensated partly by a (max 2×) longer irradiation time and a higher intensity from the accelerator. The shape and depth of the target volume will have a significant effect on this problem. The effect of the intensity reduction due to transmission losses will be more negligible if more high‐energy protons are needed, for example, in cases of deep lying “thin” targets. Another method to reduce transmission losses is to perform the energy modulation just before the patient, using a range shifter or a system analogous to an energy modulation wheel. However, in this situation, the dose from secondary particles needs to be accounted for in the delivered dose.

Therefore, an isochronous cyclotron can undoubtedly be used for FLASH experiments and, in some cases, also for clinical FLASH treatments. However, a faster energy spread and less beam‐transmission losses at low energy are required to obtain a similar distribution of energies in the target.

### 5.6 Applicability of synchrocyclotrons for UHDR irradiations

In a synchrocyclotron, the typically extracted average beam intensity is in the order of several nA, for ∼10 µs RF mod‐pulses at a PRF of 1 kHz; an average beam intensity of 10 nA is equivalent to 0.01 nC per RF mod‐pulse.

For a typical FLASH experiment discussed above, the 0.5 nC needed to deposit 2 Gy in 1 cm^3^, thus needs 50 RF pulses. At 1 kHz RF‐modulation PRF, this will take 50 ms. In such an experiment, this yields an average dose rate of 40 and 6000 Gy/s during the RF mod‐pulses. With such a synchrocyclotron, one will be thus able to perform FLASH experiments in cm^3^ sized targets. However, one should realize that it is not known from the radiobiology side whether the ∼1 ms waiting time between the RF mod‐pulses would affect the FLASH‐relevant processes. More data are needed to understand whether this is important. Furthermore, there is a technical issue to consider, especially when high intensities per RF mod‐pulse are used, that is, beam size‐dependent saturation effects will yield too low signals from the typical ionization chamber‐based dose monitors. This needs a dedicated calibration procedure or the use of other monitor types.

For clinical targets of 1 kg, the 2 Gy dose, deposited by approximately 30 nC, can be given in 3000 RF mod‐pulses, which takes 3 s. This yields a dose rate of 0.67 Gy/s during a time interval that is 30 times longer than the 100 ms commonly assumed time for a FLASH pulse. In addition to that, as with the isochronous cyclotron, the eventual intensity and time losses due to energy modulation must be considered.

So, for the example of clinical FLASH applications considered here, the synchrocyclotron will be of very limited use, with targets <0.03 kg. However, for FLASH experiments with small targets, synchrotrons can be used in most cases.

### 5.7 Applicability of synchrotrons for UHDR irradiations

#### 5.7.1 Use of synchrotron X‐rays

Since the beginning of particle therapy, synchrotrons have been used to accelerate the ions or protons. In synchrotrons accelerating electron beams, synchrotron irradiation is created due to the bending of an electron beam within the ring. Its energy spectrum and intensity differ from X‐rays conventionally produced by electrons hitting a target. Synchrotron radiation is used for various experiments in dedicated facilities, such as the ESRF in France, and SLS in Switzerland.

Due to its much higher intensity, compared with X‐ray beams from a medical linac, synchrotron radiation is of great interest for FLASH experiments. Recently, the first photon FLASH experiments have been performed at ESRF.^39^ In these experiments, 10 Gy were deposited in a mouse brain (<1 cm^3^) by synchrotron X‐rays of 102 keV. The dose was applied with a dose rate of 12 000 Gy/s in a 50 µm thick slice, through which the head of the mouse was shifted within 0.27 s. This resulted in a *DR*_ave_ of 37 Gy/s. The study showed lower brain toxicity in FLASH irradiations than conventional dose rates, making these synchrotron‐X‐ray dose applications very interesting for the study of FEs. For clinical applications, the X‐ray beam sizes currently available with synchrotrons are not sufficient.

#### 5.7.2 Use of proton beams from synchrotrons

The proton beam application of synchrotrons is also considered here regarding the possibility of using these proton beams for UHDR irradiations.

Synchrotrons get their beam from an external source, which can also make a very high intensity beam. However, the maximum number of protons injected into the ring is limited to 1–100 nC (machine‐dependent), due to space charge effects. Applying a field at the patient needs typically 1–3 spills, including a dead time of several seconds. Therefore, fewer spills, enabled by a higher ring filling, would considerably reduce irradiation time. Different groups are investigating several methods to increase the ring filling capacity to decrease the defocusing space charge effects at high ring fillings. These methods are based on higher injection energy and an extension of the bunch lengths in the ring.^40^

Another method to shorten the irradiation time is to increase the ramping speed of the synchrotron magnets.^41^, ^42^ Currently, the extracted beam intensities are in the range of 0.1–10 nA.

When using a synchrotron, FLASH experiments are possible at all beam energies. The ring can be filled with the 0.5 nC needed for the FLASH dose in 1 cm^3^. With an extracted beam intensity of <10 nA, the 2 Gy dose is applied within the usually required FLASH pulse length of 100 ms, with a 40 Gy/s dose rate. Less beam intensity is needed at lower energies due to the higher stopping power of the lower energy protons.

For the example of clinical use, the 30 nC required for the 2 Gy dose is currently at the upper limit of the possible ring filling. However, if this can be achieved, the required dose rate of 40 Gy/s is easily reached if extraction is done in a few hundred thousand turns, which takes less than 100 ms. This rough estimate has neglected eventual time and intensity losses due to energy modulation, as with the cyclotrons.

Therefore, a synchrotron can certainly be used for FLASH experiments, and in many cases, it is expected that it can also be used for clinical FLASH treatments.

### 5.8 Different beam energies in UHDR irradiations

Proton beam energy variations are needed to distribute the Bragg peaks in depth over the target. Because the beams extracted from medical cyclotrons have a permanently fixed energy of, for example, 230 or 250 MeV, beam energy must be set to the different energies in the downstream beam line. In the cyclotron systems used at present, this is done with a so‐called degrader. A degrader is an adjustable amount of material, which can be inserted into the beam line. Degraders are commonly used just past the cyclotron beam extraction but may also be used proximal to the irradiation target. The amount of inserted material determines the decrease of the proton energy. All subsequent magnets in the beam transport system must adapt their strength accordingly, limiting the energy change speed to 50–100 ms for a 2% step change in energy.^43^ In addition, in the beam transport system, following the degrader, there is an energy‐dependent beam transmission loss of more than 99% at 70 MeV.^44^ This energy‐dependent intensity variation must be taken into account.

The energy can be set per spill in a synchrotron, and the beam emittance and intensity are almost constant for all energies. However, each energy needs another spill. The energy modulation can be performed with the “classical” energy modulation wheel in the nozzle, just before the patient. However, a significant improvement has recently been achieved by enabling a slight reduction of the energy of the extracted beam within a 100 ms step, during the beam‐extraction phase.^45^ This so‐called multienergy extraction can reduce irradiation time by 30% in synchrotron facilities. Although it does not yet enable a higher beam intensity in the ring, the energy‐independent intensity of the extracted beam is very beneficial. It is not yet clear if and how this can be combined with fast extraction (in fewer turns) to achieve the high dose rate in the extracted beam.

As may be clear from above, energy variations are a limiting factor in the short time that the FLASH dose has to be applied. In current facilities, a change of energy requires at least 100 ms per energy step. This applies both to the degrader‐based systems in cyclotron facilities and synchrotron facilities using multienergy extraction. In addition, in cyclotron‐based facilities, one must deal with the energy‐dependent beam intensity at the target or patient.

Currently, the only method compatible with FLASH irradiation is to perform energy degrading just before the patient. This will also reduce beam intensity, due to lateral beam spreading, but this will be less than the losses in the beam lines, as in the current layouts. The mechanics of the degrader (or range shifter) should be made to change the energy within a few milliseconds. Alternatively, one should use a system similar to the “classic” method (in proton therapy), based on the so‐called range modulation wheel. However, the full beam is degraded over its full lateral width to the same specific energy range in such systems. A passive system could also be used. The passive system most frequently used is a ridge filter, which gives a dose range variation that does not depend on the lateral direction. A more complex method uses a filter made of cones, enabling a lateral variation of the range and range spreading, similar to PBS.

In general, such passive systems must be mounted in the nozzle because most current beam transport systems do not accept such an amount of energy spread in the beam.

## 6 LINEAR UHDR ACCELERATORS

**FIGURE 3:**
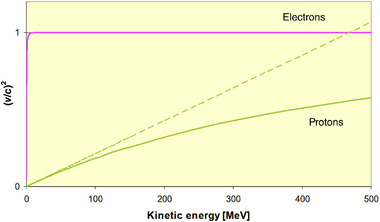
Relativistic particle velocity (squared) as a kinetic energy function (protons and electrons). Reprinted with permission from Vretenar.^46^ The dashed line represents the nonrelativistic classical relation

In contrast to circular accelerators, linacs accelerate electrons or ions in a single pass along a straight path. Consequently, the relativistic situation for linac design is much different for electrons and ions, including protons. Figure 3 shows the results of plotting *β* ^2^ as a function of energy for electrons (*m*_0_ *c* ^2 ^= 511 keV) and for protons (*m*_0_ *c* ^2^ = 938 MeV), as previously described by Vretenar.^49^

The electrons (upper curve in Figure 3) come close to the speed of light after a few MeV of energy, corresponding to the first meter of acceleration. For the remainder of the acceleration process, their velocity will not change. Also, because the electron velocity closely matches the RF speed (*c*), the electrons can be carried with the RF in a traveling wave (TW) accelerator of repeated, identical cavities.^46^

Protons (lower curve in Figure 3), in contrast, increase velocity slowly and non‐relativistically up to about 40 MeV. Above this energy, the proton mass begins to increase relativistically, and the velocity increases more slowly until *v*∼*c* above a few GeV, much higher than the energy needed for therapeutic applications, including FLASH‐RT. Therefore, unlike an electron linac, a proton (and heavier ions) linac must comprise differing accelerating cavities. Furthermore, the cavities must be optimized for the maximum energy transfer to the ions, changing from the classical to the relativistic regimes. Also, because the ions are significantly slower than the potential electric field propagation, standing wave (SW), as opposed to TW, designs are typical, resulting in a pulsed beam.

### 6.1 Electron linacs

The accelerating structure in an electron linac is composed of a multicell resonant cavity, typically made of copper, in which 3 GHz RF waves accelerate periodically, with the electrons concentrated in short bunches at the frequency of the electromagnetic wave. The electrons are accelerated when the axial component of the electric field is maximum. The length of each cell is such that the electron's motion is synchronized with the oscillation of the electromagnetic field. For a typical electron linac with a phase advance per cell of 180°, and electron traveling at the speed *β* = *v/c*, the synchronism conditions are obtained when the length of the cells is *L* = *βλ/2*, where *λ* is the wavelength in the vacuum of the RF field (typically *λ* = 10 cm).

#### 6.1.1 Clinical linacs

Clinical linacs have become standard equipment and the true “work horses” of modern radiation therapy facilities. The accelerating process happens inside the accelerating waveguide, utilizing energy transfer from the RF field distributed among the evacuated waveguide cavities to the electrons passing through. Electrons accelerated to more than 99% of the velocity of light are then brought into the linac head in the form of a pencil beam. The head is equipped with X‐ray conversion targets, flattening and scattering filters, dual TICs, and collimation systems, which all contribute to the final formation of the clinical electron and photon beams. The conversion target and flattening filters are removed in electron beam therapy, and accelerated electrons are directly applied to the patient. Because of their weak penetration in tissues, electron beams are used to treat cutaneous and subcutaneous lesions and for intra‐operative radiation therapy (IORT). Treatments of deep‐seated targets require the use of MV photon beams, which are produced upon the bombardment of the X‐ray target (usually tungsten) with the electron pencil beam. After production, photon beams can be flattened and collimated with multileaf collimators (MLCs) to achieve the needed dose conformity.

Modern radiotherapy linacs can accelerate electrons up to 18–20 MeV within 1.5 m, and they are mounted on rotating “gantries” (mechanical supporting structures). Both SW and TW types are used. The RF power sources are either klystrons or magnetrons. Klystrons are RF amplifiers, where the velocity modulation of electrons amplifies RF signals by more than 50 dB, reaching (in pulsed mode) MW level of peak power. Magnetrons are RF oscillators, where electrons emitted by a hot filament (cathode) travel outwards immersed in a magnetic field that causes them to move in a spiral path, thereby exciting RF fields in the resonant cavities’ anodes placed around the filament.

Regarding the dose rates (*DR*_ave_), current clinical linacs operate in the range of 1–10 Gy/min. Without special modifications, photon beam dose rates can reach a maximum of several tens of Gy/min in a flattening filter‐free (FFF) configuration. In contrast, an order of magnitude higher dose rates is feasible in the electron beam mode. These values are still too low for observing the FE but can be significantly improved by straightforward and reversible linac modifications.^23^, ^24^ Modified devices are suitable for preclinical FLASH investigations with UHDR electron beams. Unfortunately, UHDR conditions remain inaccessible for photon beams that suffer from the electron‐photo conversion process. See additional details in the next section.

#### 6.1.2 Modifying a clinical linac for preclinical UHDR investigations

Two groups have reported their work on modifying standard clinical linacs, Varian Clinac 21E and ELEKTA Precise, to deliver UHDR beams with characteristics similar to FLASH‐validated electron beams.^24^, ^47^ In both cases, modifications included removing the physical photon conversion target and tuning the structures that control the beam, such as the pulse forming network, electron gun, and beam steering. The UHDR conditions were established only in the electron beam mode at a small source to surface distances (SSDs), which require positioning the biological targets inside the linac head. The main drawback of such geometry is the reduced size of the radiation field. Lower penetration of electrons than photon beams, and the need to fit the biological target into the head, limit the applicability of the achieved UHDR beams to small animal preclinical studies. After tuning, the Varian Clinac reached the highest average dose rate of 900 Gy/s and a *D*_pulse_ of 8.5 Gy for a 90% beam diameter of 1.2 cm.^23^ Beam parameters were also tested at two additional positions having larger SSDs with the reported *DR*_ave_ (*D*_pulse_) of 220 Gy/s (2.5 Gy) and 70 Gy/s (0.65 Gy). At the same positions, the 90% diameter varied from 4.1 to 6.9 cm, respectively. In the subsequent experiment, the tuned beam with *DR*_ave_ of 220 Gy/s was validated for the FE in mice.^8^ Similar beam performance was obtained for the modified ELEKTA Precise machine.^24^ The authors specified *DR*_ave_ of 120, 250, and 1000 Gy/s at three different positions in the head, accompanied by *D*_pulse_ of 0.64, 1.3, and 5.1 Gy, respectively. Until now, this UHDR beam has not been FLASH validated.

Finally, the Italian company Sordina IORT Technologies transformed their Intra IORT mobile linac NOVAC7 into a UHDR compatible research machine.^48^ This transformation was more straightforward than the two examples described above and only modified the collimation system of the standard NOVAC7 machine. Again, the SSD had to be significantly shortened, which resulted in dramatic shrinkage of the radiation field. The beam was characterized at two positions, measuring *DR*_ave_ (*D*_pulse_) of 540 Gy/s (18.2 Gy) and 117 Gy/s (3.9 Gy) for electron fields having FWHM of 0.5 and 1.5 cm, respectively. No attempts have been made so far to validate this beam for the FE.

#### 6.1.3 Linear UHDR electron systems

The following electron linacs were dedicatedly manufactured to deliver UHDR beams without any modifications.

##### Kinetron

Kinetron (CGR‐MeV, Courbeville, France) is an experimental prototype linear accelerator, at the Institute Curie in Orsay, capable of generating UHDR pulsed electron beams with a nominal energy of 4.5 MeV.^49^ The machine is powered by an S‐band (3 GHz) magnetron and delivers variable *DR*_ave_ ranging from Gy/min to thousands of Gy/s. The pulsed beam structure comprises 0.1–3 µs pulses delivered at frequencies between 10 and 200 Hz. The maximum *D*_pulse_ available with Kinetron is 60 Gy. However, in most FLASH studies, the irradiator was operated with pulses carrying a dose of 1 Gy. Kinetron was used to irradiate mice in the first published FLASH‐RT study from 2014 reporting on the reduced lung fibrosis after UHDR irradiation.^2^

##### Oriatron eRT6

Like Kinetron, Oriatron eRT6 (PMB, Peynier, France) is another experimental S‐band electron linac built for Lausanne University Hospital, Switzerland. It is almost exclusively used for studying the occurrence and the underlying mechanisms of the FE in a preclinical setting.^50^ The nominal energy of the eRT6 electron beam is between 5 and 6 MeV. Its time structure comprises 0.5‐4 µs pulses, with PRF varying between 5 Hz and 200 Hz. Pulse dose can be controlled via three parameters: the tension on the grid of the electron gun, pulse width, and SSD. Usually, the grid tension and pulse width are fixed to 300 V and 1.8 µs, respectively. The *D*_pulse_ in the FLASH mode is tuned then by positioning the samples at the appropriate SSD. Values are commonly used between 1 and 10 Gy, accompanied by pulse dose rates (*DR*_pulse_) of 10^5^–10^6^ Gy/s. Changing the SSD directly impacts the beam size, which measures 9 cm (90% diameter) for a *D*_pulse_ of 1 Gy, decreasing to 4 cm for a *D*_pulse_ of 5 Gy, and further to around 3 cm for a pulse dose of 10 Gy. Oriatron eRT6 was commissioned in 2017 and has produced most of the FLASH results published in the literature. Also, the first administration of FLASH‐RT in a human patient was performed with this machine.^26^

##### Mobetron

The Mobetron (IntraOp, Sunnyvale, US) is a medical electron irradiator dedicated to IORT and treating superficial targets.^51^ A particular version of Mobetron that supports operation in two modes, UHDR and Conv, was commissioned for preclinical and clinical applications. The Conv mode is equivalent to any standard Mobetron linac operation, whereas the UHDR beam was optimized to resemble the FLASH‐validated beam of the Oriatron eRT6 irradiator. Beam energies of 6 and 9 MeV can be selected in the UHDR mode, with only the latter being available in the Conv mode. *D*_pulse_ in the UHDR mode can be varied up to 9.2 Gy achieved at a position near the beam exit window (effective SSD 17.3 cm). A beam size (90% diameter) of 3.8 cm was reported at the same position. The beam broadens, and the pulse dose decreases, at distances further away from the exit window. At the effective SSD of 37.3 cm, the maximum *D*_pulse_ was 3.3 Gy, whereas the 90% beam diameter measured 6 cm. The highest available PRF is 90 Hz.

##### VHEE accelerators

Reistad and coworkers^52^ developed a 10 MeV electron beam accelerator for radiotherapy from microtron technology developed by Veksler,^53^ which was later improved for producing beams with energy up to 100 MeV. In parallel to microtron technology, the electron linac approach has grown and, due to its relative compactness for up to 20 MeV energy range, appears more suitable for implementation on a gantry, thus diminishing the interest in microtrons for radiotherapy. In a typical linac, the maximum electric field is limited to about 1 MeV/cm. Above this limit, the accelerating cavity gets ionized, and the electron beam is no longer produced in a controlled way. For this reason, only electrons in the 7–20 MeV energy range are used in radiotherapy, which is only for IORT and superficial irradiations.

**FIGURE 4:**
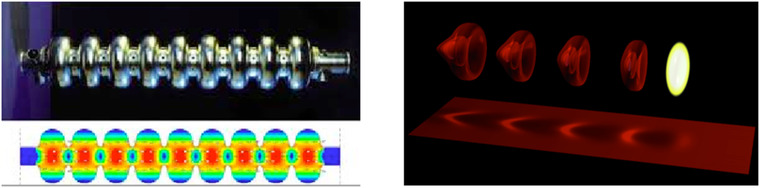
Accelerating structures producing few tens of MeV gain. Left, 1‐m‐long radiofrequency (RF) cavity, shown in the top image, with the corresponding electric field map shown below. Right, 100 microns long plasma “cavity” created by the laser pulse (shown in yellow) which propagates to the right

This limited range of electron energy of typical medical accelerators does not allow the treatment of deep‐seated tumors. Moreover, the large electron lateral penumbra may often be prohibitive for an adequate dose conformation to the target volume inside the patient. This problem can be solved by increasing the electron energy to values greater than 50 MeV. In that case, the penetration distance is deeper, the transverse penumbra becomes steeper, but the fall‐off distance increases. The advantages of VHEE beams for clinical purposes were investigated at the beginning of this century.^54^, ^55^ Isodose simulations in the treatment plan with VHEE in prostate cancer indicate a better target coverage with a better sparing of normal tissues than the conventional photon intensity‐modulated radiation therapy (IMRT). A detailed study of the dosimetric properties of monoenergetic VHEE beams in the range of 150–250 MeV shows, for instance, that for parallel opposed beams, the sharpness of the lateral penumbra is of comparable quality to that of clinical photon beams.^56^ These results have triggered projects in Europe and in the USA to develop VHEE with advanced RF cavities. Examples of an electron linac and proton accelerator intended for VHEE are presented in Figure 4.

Recently, the Conseil Européen pour la Recherche Nucléaire (CERN) has proposed electron accelerator technology, based on its Compact Linear Collider (CLIC), that may provide electron UHDR to tissue depths of 20 cm. The CLIC‐based delivery system uses high‐gradient “X‐band” RF and is intended to provide conformal UHDR deliveries, as well as VHEE transmission beams. The CERN system is estimated to have maximum electron energy of about 100 MeV.^57^ Together with CERN, the Centre Hospitalier Universitaire Vaudois regional Swiss hospital intends to build a VHEE prototype, based on the CLIC technology, capable of UHDR for preclinical and clinical use.

### 6.2 Proton UHDR linacs

Proton linacs have long been developed at CERN.^58^, ^59^, ^60^ Based on this development, Advanced Oncotherapy plc (AVO), UK, a CERN‐spin off company, integrates a series of SW linac structures into their LIGHT (Linac for Image Guided Hadron Therapy) system, providing it for proton therapy.

The LIGHT proton acceleration is provided by the axially and linearly accelerating gradient energized by a set of klystron/modulators operating in the S‐band at 2.998 GHz. The maximum accelerating gradient achieved is about 18–20 MV/m, resulting in 230 MeV acceleration in 24 m, including the source and lower, constant energy (<37.5 MeV) sections. Also, the proton source is “chopped” at 200 Hz, providing a proton pulse every 5 ms with about a 2 µs duration, both considered as beam macrostructure. The 2 µs pulse contains 749 Hz and 2998.5 GHz microstructure within the linac accelerating cavities,^61^ as described in additional detail below.

**FIGURE 5:**
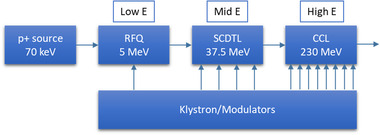
Schematic layout of the LIGHT beam delivery system

In general, linac cavity‐type choice depends on particle type, energy, beam current (*I*), duty cycle (pulsed, CW), frequency, cost of fabrication, and operation.^62^ The LIGHT proton linac utilizes three different types of cavities for maximum proton energy transfer efficiency. As shown in Figure 5, the LIGHT system uses a RF quadrupole (RFQ), four side‐coupled drift tube linacs (SCDTLs), and 15 charge‐coupled linacs (CCLs).

The different types of linac cavities are used to optimize the power transfer efficiency. The power transfer efficiency is characterized by a linac cavity's shunt impedance specified per unit length (MOhm/m). Ideally, the shunt impedance should be ample for the accelerating mode, so that the dissipated power is small. This is particularly important for copper cavities, where the wall power dissipation is a major issue, and it is desirable to have as large an accelerating field as possible.^63^

**FIGURE 6:**
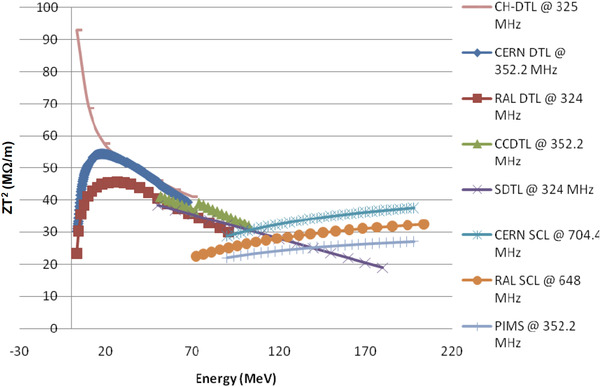
Relation of shunt impedance per unit length with proton energy. Higher shunt impedance provided more efficient energy transfer to the accelerating protons. Adapted with permission from Plostinar.^64^

The shunt impedance depends on the ion type, ion energy, frequency, and cavity type. Figure 6 shows that the RFQ (labeled CH‐DTL; CH, cross‐bar H‐type), SDTL (S, side), and CCL (labeled PIMS for PI‐mode structures) provide optimal shunt impedance over the ranges of 0.1–5, 5–37.5, 37.5–230 MeV, respectively.^64^

#### 6.2.1 RF quadrupole

The RFQ operates at a quarter of the S‐band frequency, 749 MHz. Overall, the RFQ focuses, accelerates, and bunches the protons. The RFQ acceleration is 5 MeV in only 2 m; it is divided into four standardized modules of 500 mm, each equipped with 12 tuner ports and one RF input. The inner quadrant radius is 46 mm, and the RFQ has an outer diameter of 134 mm; its total weight is only 220 kg. The beam dynamics and RF design have been optimized for reduced length and minimum RF power consumption.

#### 6.2.2 Side‐coupled drift tube linac

Operating at 2.998 GHz, the four SCDTLs provide proton acceleration to 37.5 MeV in 6.2 m. The SCDTL structure consists of short DTL tanks coupled together by side cavities. The drift tubes provide zero‐field regions for the protons to traverse, where the applied electric field is reversed using the *π*/2 mode. The side cavities of the SCDTLs allow the length of the drift tubes to be minimized. Therefore, the DTLs are short tanks, each having 6 cells of *βλ* length, and the side cavity extends in a space left free on the axis for the accommodation of a very short (3.3 cm long, 2 cm outer diameter, 7 mm inner diameter) permanent magnet quadrupole (PMQ) for transverse focusing.

#### 6.2.3 Charge‐coupled linac

High energy, relativistic acceleration is efficiently provided by the CCLs, achieving 192.5 MeV energy gain to a total of 230 MeV in 15.5 m (for the CCLs). In addition to acceptable shunt impedance at higher energies, the CCL cavities permit PMQs between the 15 CCLs, enabling a reduction in length of the DTLs and thus increasing the RF efficiency. Therefore, the DTL diameter is small, about 4–5 mm, maintaining high RF efficiency and permitting precise PMQ alignment independent of the physical cavities.

#### 6.2.4 High pulse rate proton linac

**FIGURE 7:**
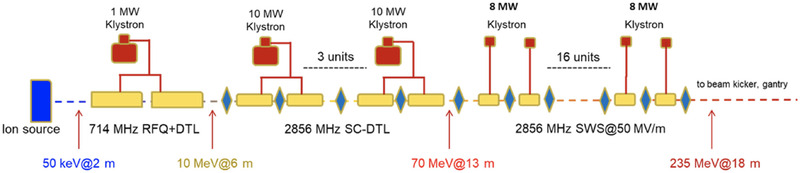
Schematic design of compact UHDR linac. Reprinted with permission from Fang et al.^65^

Recently, researchers reported on a 1000 Hz high pulse rate proton linac designed for UHDR.^65^ As shown in Figure 7, the UHDR linac is based on the AVO LIGHT system with some modifications: higher gradient linac structures, higher source current, five times greater pulse frequency, and five times longer pulses.^65^

The high‐gradient linac structures do not increase the beam current but result in a slightly more compact design, that is, 18 versus 24 m for LIGHT. However, high‐gradient structures use more power and are more susceptible to arcing. The authors argue that the power consumption will be reduced because of the short FLASH deliveries, but this critical performance choice may prevent use for non‐UHDR proton therapy. Serving its performance objective, the authors claim the system is capable of *DR*_ave_ of 30 Gy within 100 ms, implying 100 pulses of 0.3 Gy *D*_pulse_. Here, we note that *D*_pulse_ may be a critical FE parameter for ion linacs as with electron linacs. The design also depends on a novel beam delivery system (BDS), see Section 8.

#### 6.2.5 SC linacs

**FIGURE 8:**
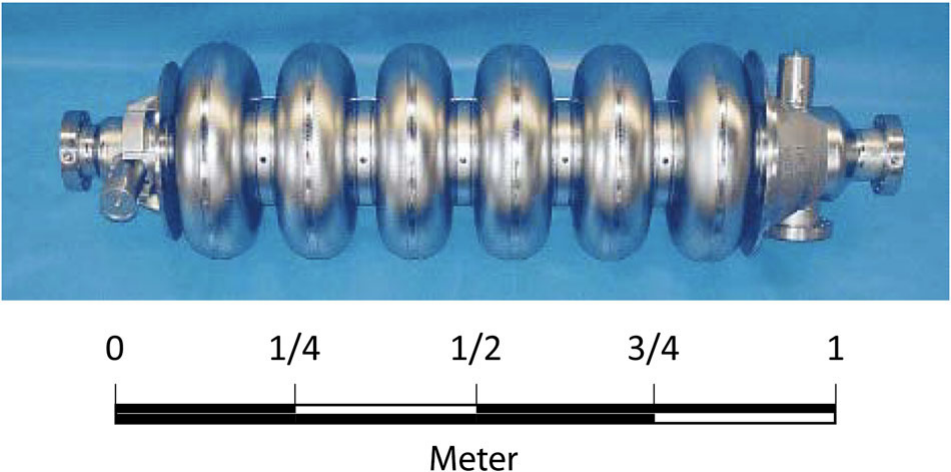
High beta 6 cell 805 MHz cavity. The series production for the SNS described by Pekeler et al.^66^ fabricated 74 of the high beta cavities

Typical accelerator design driving aspects are the desired voltage, the duty factor of accelerator operation, beam current, or beam power. Higher beam current designs may consider CW designs. As example, consider the pulsed SC linac of the spallation neutron source (SNS) at Oak Ridge, USA. Using six‐cell niobium cavities^66^ (see Figure 8) at 804 MHz, the SNS produced a proton beam from 200 to 1000 MeV to put out mA order of magnitude current.^67^

For CW ion cavities, SC technology can be considered to reduce power needs. The need is increased for shorter, higher gradient structures. This is because the losses in the accelerating cavity walls increase as the square of the accelerating voltage. Hence, normal conducting copper cavities become less effective as the need for high continuous‐wave (CW) voltage grows with particle energy. However, the RF surface resistance of a superconductor is five orders of magnitude less than that of copper. A SC resonator's quality factor (*Q*_0_) is typically billions (i.e., a billion oscillations before the resonator energy dissipates). After accounting for the refrigerator power needed, the overall cooling power's net gain remains a factor of several hundred. Indeed, the higher‐voltage, shorter SC structures can also reduce the disruptive effect accelerating cavities have on the beam. The reduced beam perturbations can result in better beam quality, higher maximum current, and a smaller beam halo, thereby reducing the machine radioactivation.^63^

#### 6.2.6 Linac booster

Linac cavities have also been considered for the application of increasing (boosting) proton beams produced by cyclotrons. The hybrid approach offers the advantage of high current injection from low energy, compact cyclotrons, while retaining the attractive, fast energy change property of linacs. However, the beam coupling between a cyclotron and linac is complicated by the significant differences in the emittances of the two types of accelerators. A LInac BOoster (LIBO) CCL structure has already been designed and tested.^68^, ^69^ LIBOs have also been considered to boost the energy of a cyclotron‐based proton therapy system for proton imaging.^70^, ^71^

### 6.3 Optimizing proton linacs for UHDR

The quantity of beam output (I) of a linac can be optimized through design. The design factors related to linac output are presented and discussed in this section. Specifically, Table 4 lists the parameters and design choices to be considered to produce UHDR proton beams from a linac. The potential UHDR developments are split into two categories: pulse rate, pulse length, and source updates to existing SW accelerators; or a transition to TW, CW, and SC technology. Arguably, further UHDR of SW linacs, such as LIGHT, will be more easily achieved than other linac types.

**TABLE 4:** Design parameters of a proton linac for UHDR

Attribute	UHDR factor increase	Comments
Frequency (*f*) [Hz]	∼*f*	Ion linacs usually operate 200–400 Hz. Frequencies up to 1000 Hz are under consideration for UHDR.
Pulse length (*L*) [µs]	∼*L*	Longer pulses contain more protons up to the transmission limit
Source current (*I*) [mA]	∼*I*	The output is linear with the source current up to the space charge limit and acceptance by the RFQ.
Accelerating potential [MV/m]	None	Ultra‐high dose rates do not depend on the accelerating potential of a linac.
Superconducting	Many times, up to mA	Controls cavity overheating, enabling CW, and provides efficient current distribution.
TW, CW	Many times	TW can “push” more current but may be a challenging design for ions. CW with superconducting.

## 7 LASER UHDR ACCELERATORS

**FIGURE 9:**
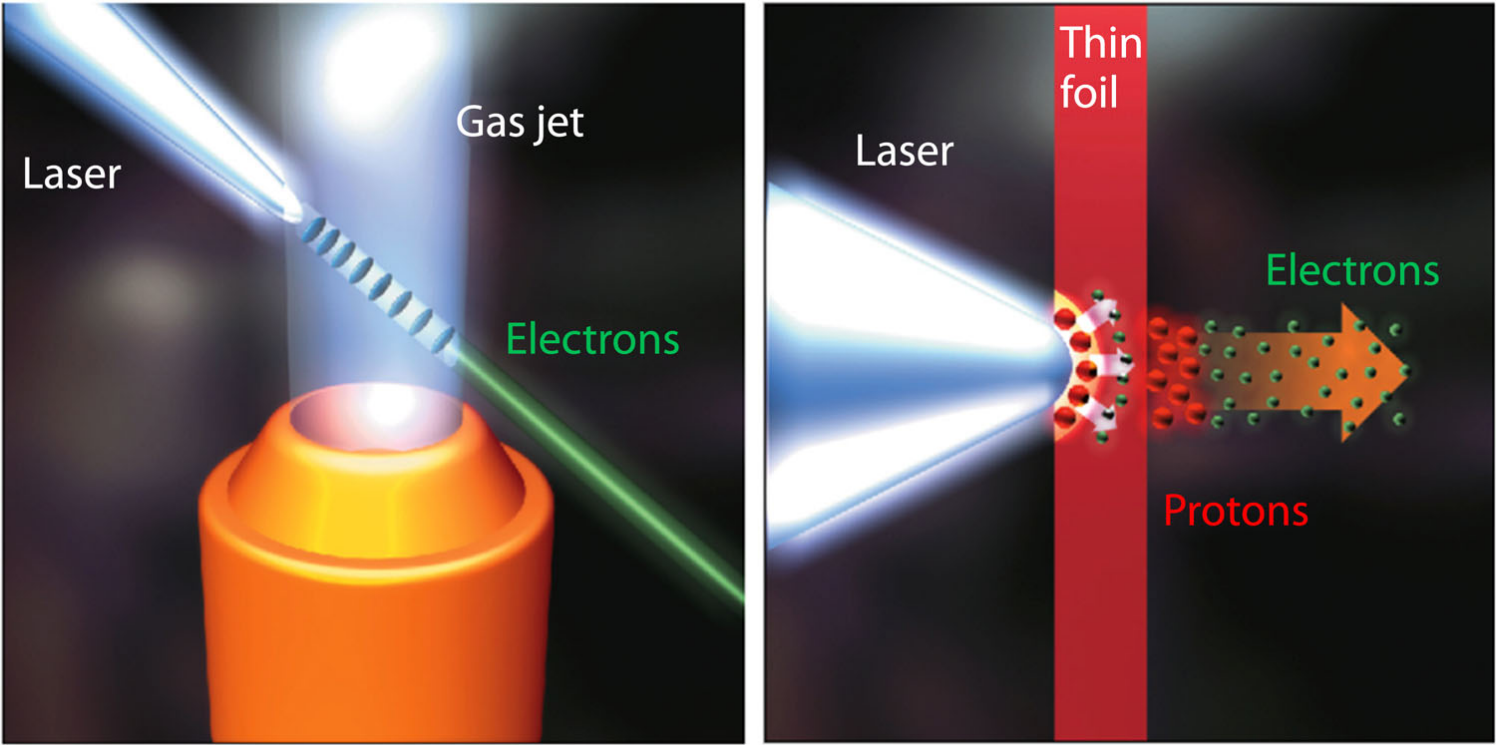
Principle of laser‐plasma accelerators. Electron wakefield acceleration in millimeter long gas target (left), and proton acceleration from micrometer thick foils (right). The laser beam (in white color) that propagates from left to right produces after interacting with the target collimated beams of energetic particles

The coherence properties of the laser light allow the concentration of the laser pulse energy into an extremely small volume. Using a few femtoseconds duration (a few micrometers pulse length), laser beams are easily focused down to a focal spot with a radius of a few micrometers, allowing the laser pulse energy to be contained in a sphere with a radius of a few micrometers. The target materiel is suddenly ionized when interacting with a thin foil or a gas jet (Figure 9). The collective motion of electrons efficiently transforms the laser's ultraintense transverse electric into an accelerating longitudinal field with peak amplitudes that can exceed a TV/m (10^12^ V/m) range. This can provide more than three to four orders of magnitude larger output than conventional accelerating field values, such as those used in RT machines, motivating scientists to develop a new “plasma” based technology for accelerators. Laser‐plasma accelerators (LPAs) for RT with VHEE or proton beams are among these developments.

Interestingly, the duration of these beams being extremely short (tens of femtoseconds for electron and a few picoseconds for proton beams) could, if not satisfying the FLASH requirements, contribute to extending innovative research on the effects of UHDR on cells. LPA beams might produce 1 Gy in a single femtosecond pulse at 10 Hz PRF, up to tens of Gy in a single pulse at a 1 Hz PRF. This enables a wide range of *D*_pulse_ for FLASH studies.

### 7.1 Laser UHDR VHEE

In 2002, a LPA (see Figure 9 for an illustration of the principle) delivering a VHEE beam at 200 MeV with world‐record electric field values in the hundreds of GV/m (10^9^ V/m) was demonstrated.^72^ In the following years, significant breakthroughs, including the “dream beams” with quasi‐monoenergetic electron distribution,^73^, ^74^, ^75^ were demonstrated, and just after that, a stable monoenergetic electron beam with adjustable parameters (such as electron energy and charge) was demonstrated.^76^ These breakthroughs show the potential of LPAs for societal applications^77^ and have triggered more exploration towards RT applications.

Today, typical working conditions of LPAs in delivering VHEE RT are a PRF of 1 Hz in routine (or of 10 Hz occasionally, because of the lifetime of the expensive gratings), tens to hundreds of pC charge in the 250 MeV energy range (within a suitable few MeV energy bandwidth), which should allow reaching about 20 cm depth, at 50 Gy in 1 min, in 1 cm^3^ volume.

### 7.2 Laser UHDR proton

**FIGURE 10:**
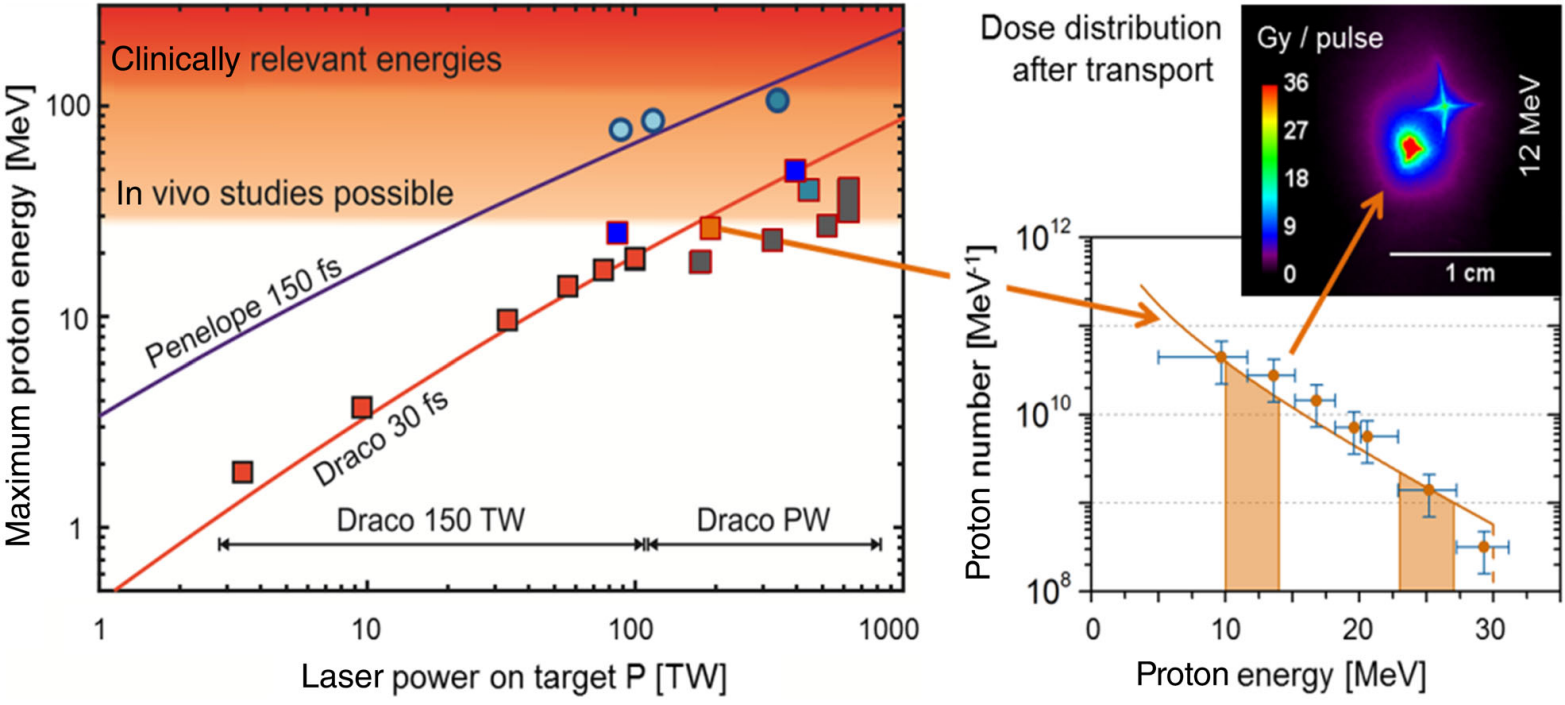
Proton energy scaling with laser power at both HZDR lasers (data points for 150 fs from other single‐shot systems for reference), with a typical exponential 30 MeV maximum energy spectrum taken at DRACO‐PW, and energy selective dose distribution on sample film stacks (on the right) after pulsed solenoid magnet beam transport. Courtesy of Prof. U. Schramm, Institute for Radiation Physics and Oncoray, Helmholtz‐Zentrum Dresden‐Rossendorf, Germany

Since the first acceleration of tens of MeV protons in 2000, LPAs have attracted considerable interest as an alternative to medical cyclotrons because of their compactness and cost reduction. In contrast to electrons, protons are accelerated in a nontraveling wave, corresponding to the TV/m electric field at target interfaces irradiated by the intense laser pulse. These fields originate from the expansion of energetic electrons efficiently heated by the laser pulse when interacting with the target. The power‐law dependence of the energy of the protons with the laser intensity (see Figure 10), with an exponent coefficient compromise between 0.5 and 1, depending on laser and foil conditions, shows the need for a laser capable of a few PetaWatts (PWs) to reach 200 MeV energetic protons. With the increasing number of laser beam facilities delivering hundreds of TW up to a few PWs, the progress in laser proton acceleration has shown a rapid evolution, with nearly 100 MeV proton beams produced by a PW class laser system. Laser technology is progressing significantly, making available reliable machines that routinely deliver laser pulses of PW level in 1Hz repetition rate. The laser beam parameters (e.g., spatial and temporal) are now well controlled. By satisfying most of the requirements for producing high repetition rates, and reliable and energetic proton beams, the laser‐based approach is promising for FLASH studies using tens of MeV protons. The very high proton number per pulse (see Figure 10) already deliverable by laser proton accelerators confirms their pertinence for FLASH investigations.

Radiobiological efficiency, measured under conditions similar to conventional schemes (accumulating multiple pulses over several minutes, typically delivering a few hundred mGy/pulse of intense bunches of laser‐accelerated protons), does not indicate significant differences from conventional irradiation.^56^, ^78^

### 7.3 Laser‐proton hybrid

Hybrid machines such as the Laser‐hybrid Accelerator for Radiobiological Applications, described by Aymar et al.,^79^ combine LPAs and conventional accelerators. In this configuration, the LPA first delivers the high dose rate proton beams, with protons energy in the 10–15 MeV range, that is, energies large enough to avoid the space charge effect. The proton beam is then efficiently captured in a plasma lens, before being accelerated in a conventional fixed‐field accelerator to reach higher energies of 127 MeV. With a 1 mm beam size irradiation, an instantaneous dose rate (*DR*_pulse_) of a few 10^8^ Gy/s, and a *DR*_ave_ of 150 Gy/s, are targeted.

### 7.4 Laser UHDR electron delivery

LPAs deliver collimated VHEE beams with a typical charge per energy bandwidth of about 10 pC/MeV. These beams are easily produced with a 1 J laser system working at a 1 Hz repetition rate and are expected to be delivered soon at 10 Hz. Depending on the modus operandi, beams with charges varying from 0.01 to 1 nC are achievable. The typical bunch duration is in the femtosecond range, and considering a few mm^2^ irradiation areas, the corresponding *DR*_pulse_ ranges from 10^10^ to 10^12^ Gy/s. In contrast, the *DR*_ave_ is about a few Gy/min.

VHEE beams with a higher charge/dose per shot can be obtained with a more energetic and costly laser working at a lower repetition rate. A few pC electron MeV beams are also being produced with a mJ class laser system operating at a kHz repetition rate, opening opportunities for FLASH delivery with few MeV/fs electron pulses.

### 7.5 Laser UHDR proton delivery

Protons with energies greater than 10 MeV require more energetic laser systems. They are produced primarily using two different laser technologies that enable the delivery of hundreds of TW to PW laser power. The first one is the hundreds of TW (a few J in tens of fs pulses) laser system, running at 1 to a few Hz. The second one delivers longer laser pulses in the ps range with hundreds of J of energy but at a lower repetition rate of about 1/1000 Hz. The typical charge per energy bandwidth is respectively 0.1 nC/MeV to a few nC/MeV. In both cases, the duration of the proton bunch is in the few ps time scale. Depending on the laser energy, up to a few Gy per pulse are achievable in a few mm^3^ water volumes.

In summary, laser technology is attractive for reaching UHDR for electrons and protons. Representative DR_pulse_ values for VHEE and protons are listed in Table 5. Laser technology can also provide more effective proton sources for other accelerators, including linacs (Section 6.2).

**TABLE 5:** Physical parameters of laser HUDR beams

Beam	Achieved energy	Frequency	Instantaneous dose rate (DR_pulse_)
VHEE	250 MeV (or higher)	10 Hz	Up to 10^13^ Gy/s
Protons	Up to 100 MeV	1 Hz	Up to 10^12^ Gy/s

## 8 COMPARATIVE PROTON ACCELERATOR PERFORMANCE

### 8.1 Comparative accelerator beam properties

A set of basic performance parameters can characterize each type of medical accelerator. The accelerator parameters are also related to UHDR performance. The fundamental beam properties between cyclotrons, synchrocyclotrons, synchrotrons, lasers, and linacs are compared in Table 6.

**TABLE 6:** Comparative beam properties of medical accelerators

Accelerator type → Parameters ↓	Cyclotron	Synchrocyclotron	Synchrotron	Linac	Laser
Beam emittances	3.0–9.0	3.0–6.0 Radial	1.0–2.5	0.25	0.5
(norm.) [π‐mm‐mrad]	(before ESS)	3.0–4.0 Vertical			
Energy modulation (variation)	Only with degrader‐absorbers	Only with degrader–absorbers	Possible, but slow (now multiflat‐top extraction)	By electronic control	Mechanical beam energy selector with quadrupoles
Proton losses, activation, and time structure	High in ESS	High in degrader	Small losses	Low	Medium proton losses, medium activation, short time structure
	(1/*E* dependent)		in extraction		
Change of energy (speed)	80 ms to 2.1 s	50 ms to 2 s	2–3 s	5 ms	1 s

**FIGURE 11:**
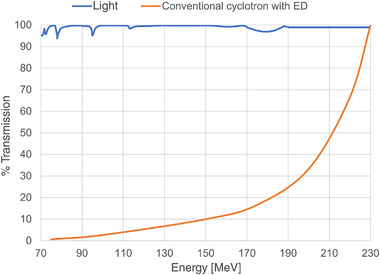
Comparison of percent transmission versus energy for a proton linac versus a conventional cyclotron. LIGHT, a proton linac; ED, energy degrader

Comparing the accelerator types, the beam properties from a proton linac are fundamentally different from those produced by circular accelerators. Moreover, each beam property can impact UHDR delivery. The proton linac beam emittance is 10× smaller than for circular accelerators. The potential to produce proton minibeams using a linac enables a locally high proton spot dose. Energy changes are electronically performed with a linac, whereas cyclotrons require thick, mechanical energy degraders (see Sections 5.8 and 9.3). The implications of the different energy selection systems are presented in Figure 11.

As shown in Figure 11, a cyclotron produces maximum output (flux) at the extraction energy, the maximum energy. At lower energies, the thick degraders reduce the energy and the beam flux until more than 99% of the produced protons are absorbed at the lowest energy, usually 70 MeV.

Because of beam reduction at lower energies, circular accelerator UHDR irradiations are typically performed at maximum energy, shooting the proton beam through the target and body. In contrast, the proton linac output is invariant with energy and could conceivably produce the same dose rate at any patient depth. Also, the linac's fast energy changes could support volumetric UHDR scanning, that may not be possible without a physical range compensator for circular accelerators.

### 8.2 Comparative accelerator UHDR performance potential

**FIGURE 12:**
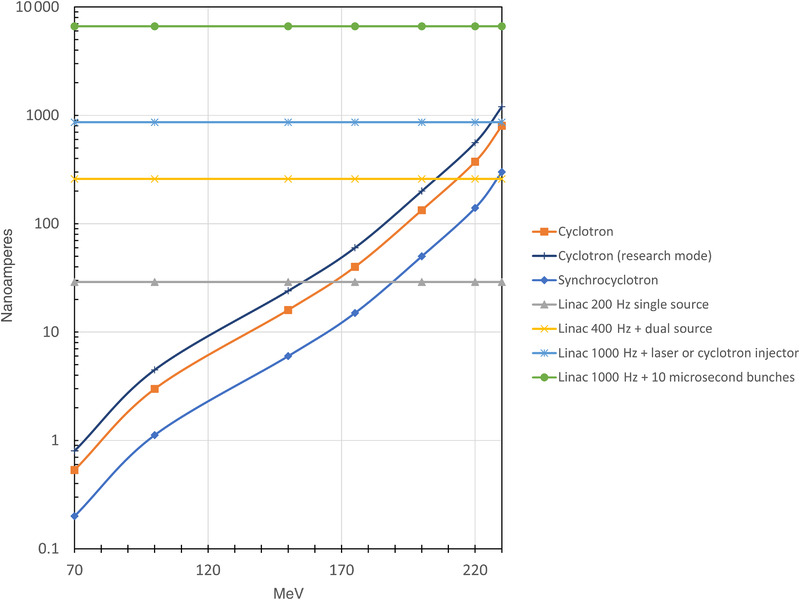
Proton beam current (average) versus energy for cyclotrons, a synchrocyclotron, and linacs

Here, we consider existing and developing accelerators for UHDR. Cyclotrons are the longest‐serving accelerators in proton therapy.^80^ In this sense, cyclotron performance is arguably the longest studied and optimized. Slow cycling synchrotrons may provide high DR_pulse_ values, but they are not sustainable in quasi‐continuous output. Laser‐based technology is attractive but has a time horizon, which is too long for clinical systems. Therefore, this analysis concerns medical cyclotrons, synchrocyclotrons, and linacs. Figure 12 compares the accelerator beam current from the available and developmental accelerators.

Of existing systems, the medical cyclotron has the highest beam current at maximum extraction energy. Reportedly, a medical cyclotron may be capable of up to 1200 nA (dark blue hashed line in Figure 12) in “research” (i.e., nonclinical) mode.^81^ Similarly, a medical synchrocyclotron (medium blue line with diamonds in Figure 12) is reportedly capable of up to 300 nA of beam current at the maximum extraction energy. These systems are currently in preclinical use, researching the FE. A similar medical cyclotron is being used for proton transmission FLASH human safety trials.^25^ However, as described in Section 8.1, the cyclotron‐based technology cannot deliver UHDR at lower energies. According to Figure 11, the cyclotron‐based system rapidly falls out of the UHDR regime below 200 MeV, far above the average energy (∼175 MeV, depending on case‐mix) needed for conformal proton therapy.

The authors have argued for conformal UHDR deliveries. These require: (1) high beam current at all energies; and (2) fast energy switching. Therefore, we consider energy invariant beam current accelerator development here. Four configurations of medical linacs are represented in Figure 12. The figure shows that, because medical linacs control energy electronically without degraders, they are all beam current invariant with energy. This is a required fundamental property to perform scanned, conformal, clinical UHDR at any depth in a patient. Having beam current energy invariance, it is a matter of scaling up the linac output to the needed UHDR value. The needed minimum UHDR value remains unknown, and we can assume development costs and time may increase with higher UHDR. Hence, several medical UHDR linac concepts are compared in Figure 12. As discussed in Section 6.2, there are a variety of approaches to increase beam current from a medical linac. First, the PRF and source current are relatively facile linac system parameters to increase, resulting in the output shown in Figure 12 by the yellow line with crosses (200 → 400 Hz, 2× source current). The modeled PRF and proton source current increases provide a medical proton linac with comparable beam current to cyclotron‐based systems, but with the vital difference of providing the needed beam current at all energies. However, ultimately, due to the space charge effect, regular proton sources are limited.

Moreover, if a higher beam current is still needed, more challenging improvements, such as laser‐based sources, may be required. Higher output sources can be combined with a higher PRF of 1000 Hz, resulting in the system represented in Figure 12 by the light blue line crossed with asterisks. Longer proton pulses (containing, by time, proportionally more protons) can be used, resulting in more than two orders of magnitude greater output (green line in Figure 12) than the base medical linac system,^65^ also well above existing medical cyclotrons.

## 9 UHDR BDSs FOR PROTONS AND OTHER IONS

The UHDR BDS provides the final beam shaping and monitoring functions. Lateral beam shaping is always needed, and longitudinal beam shaping is necessary for nontransmission proton and other ions beams. This section discusses the currently used techniques and also considers UHDR beam shaping approaches under development. The conceptualization of an ideal UHDR BDS is complicated by the lack of a complete understanding of what parameters induce, and are compatible, with the FE. Concerning the BDS, these factors include: the intensity of the beam and beam pulses; spot size; energy change time; scanning speed; and field combinations, or the lack thereof. Essentially, these are all of the requirements to design a BDS.

A significant issue for clinical applications is the uncertainty regarding whether the advantages of FLASH would allow a compromise on conformity of the dose distribution. Currently, avoiding or limiting doses in healthy tissue is more effective than what is expected to be achievable with FLASH treatments. Therefore, in the authors’ opinion, one should consider the FLASH advantages as an extra benefit and the best possible conformity of the dose to the tumor volume.

Without precise requirements for the BDS, we instead focus on discussing UHDR BDSs that can deliver as much dose, in as short a time as possible, while covering target volumes of clinical interest. We will consider potential modifications to existing systems as well as new systems under development.

### 9.1 Beam delivery angles

Traditionally, ion (including proton) treatment rooms can be equipped in various ways. In some of them, the beam is fixed and is aimed at the isocenter (usually the center of the treatment location) from a fixed direction, usually horizontally. The patient is located on a treatment table or a dedicated chair. However, in most treatment rooms, the beam is directed to the isocenter via a rotatable magnet system called a gantry. Detailed ion therapy gantry descriptions are available elsewhere.^30^ Such a gantry can rotate the last part of the beam transport system around the patient, positioned on a treatment table. The combination of gantry angle and table orientation enables many different incident‐beam directions (usually called “fields” or “beam angles”), to optimize the dose distribution in the target volume, while keeping a low dose in the surrounding healthy tissue.

There are several points to consider about providing multiple beam delivery angles and the FE. Conceivably, the time delay of changing beam angles might affect the FE. For safety reasons, mechanically rotating gantries are restricted to one revolution per minute.^82^ If we restrict the irradiation to 0.5 s and allow motion during irradiation, 3° of rotation would be possible within the allotted irradiation time. However, such minor beam orientation differences are clinically inconsequential. Ion therapy typically uses two to three beam angles, separated by 30° or more. Conceivably, if we consider the FE exclusively for nontarget (healthy) tissues we wish to spare, and the target dose rate is not a factor (i.e., it is allowed to vary), nonoverlapping beam angles may be feasible. However, to deliver an entire UHDR irradiation at multiple beam angles, an ultra‐fast, likely nonmechanical beam rotating device would be needed (see section 10.1 for examples).

### 9.2 Beam energy change time

Because, for circular accelerators, the energy is set and varied in (or immediately after) the accelerator, all magnets in the beam transport system and gantry must vary their strengths accordingly. The speed by which the field strength of magnets can be changed is limited, and consequently, the change of energy requires several hundred milliseconds up to a few seconds, depending on the system. In systems where the energy variation is done using a degrader mounted just before the patient and with synchrocyclotron systems located in the treatment room,^83^ the degraded beam does not have to pass a beam transport system. Here, the speed of energy change is determined by the mechanical speed of the degrader system, which is in the order of a few tens of milliseconds. The strengths of the fast‐scanning magnets for PBS can be corrected synchronously with the degrader setting.

In these current systems, the energy is varied in time, resulting in the Bragg peak's longitudinal shift. This will result in a variation of dose rate in the different tissues. In most cases, the distal part will receive its total dose within approximately 10 ms. Still, more proximal portions will undergo almost continuous irradiation by several Bragg peak plateaus over a fraction of a minute.

In contrast, a proton linac changes energy at its pulse rate. In conjunction with a faster beam transport system, such as a fixed field alternating gradient system,^84^ the proton linac promises 5 ms energy changes. The resulting ultra‐fast energy changes in the target offer an attractive development for scanned UHDR deliveries (see Section 9.4.2).

### 9.3 Static UHDR BDSs

Static BDSs do not contain devices that displace the beam temporally within the irradiation volume during treatment. Although UHDR dependency on the accelerator beam time structure remains, static BDSs remove the dose rate time dependency concern from the BDS part of the delivery. Static BDSs harken back to the beginning of ion therapy systems.^85^, ^86^ A static BDS is used to shape the proton beam laterally and longitudinally. Because PBS system energy changes are much slower than the lateral beam scanning speed (typically 20 m/s), a static longitudinal dose spreader might be combined with a PBS. Alternatively, a full static BDS could be considered for FLASH applications.

**FIGURE 13:**
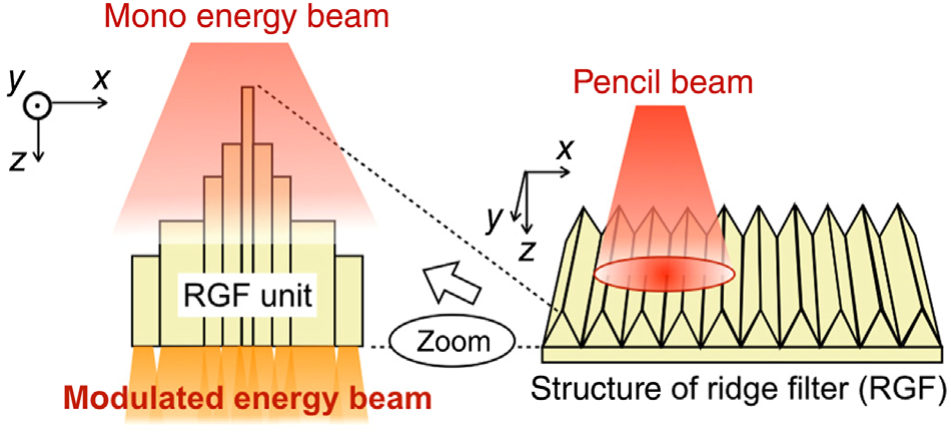
Principle of a ridge filter to spread the Bragg peak in‐depth in the patient located below the filter. Reprinted with permission from Tansho et al.^87^

Considering static longitudinal dose shaping, ridge filters, such as the one described by Tansho et al.^87^ (see Figure 13), could be applied. A ridge filter is a plate made of, for example, lucite (or another low Z material) in which almost triangular ridges are milled so that protons traversing this plate will pass different material thicknesses.^86^, ^87^, ^88^, ^89^, ^90^ Ridge filters can also be designed with arrays of holes in a low Z material.^91^ The thickness of the ridges defines the range over which the Bragg peak is spread out.

**FIGURE 14:**
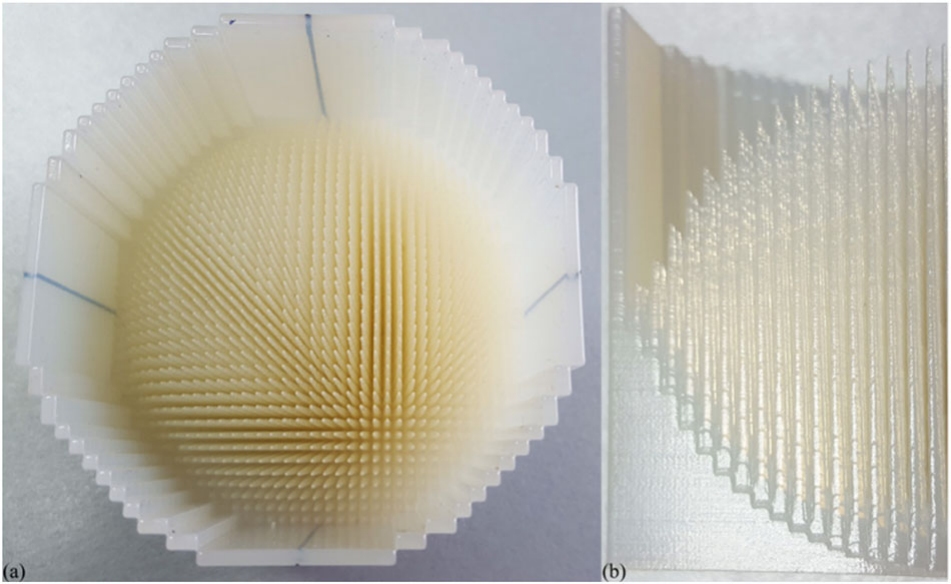
Example of a 3D range modulator; top view (a) and a quarter of it, side view (b). Diameter *d* = 5 cm, height *h* = 4.6 cm. Reprinted with permission from Simeonov et al.^92^

Conventionally, the surface of a ridge filter is covered by ridges so that one obtains the same Bragg peak spreading over the entire lateral field. This spread is similar to the energy modulation obtained with an energy modulation wheel. A modulation wheel was used in conventional scattering nozzles before PBS, but ridge filters are also used in scanning systems. In more advanced versions of the ridge filter, one could use a 2D pattern of pyramids or cones,^92^ instead of ridges, to introduce a lateral variation of the Bragg peak spreading in two dimensions (see Figure 14). The distance between the pyramid filter and the patient must be smaller than for a regular ridge filter to keep this desired correlation between energy(spread) and lateral position. With such filters, the high dose volume can be better confined to the tumor volume.

The advantage of such a filter is that the lateral PBS can be done in a single scan over the lateral surface and, since it is a passive system, there is no time‐dependent dose rate variation in the tumor volume in the longitudinal direction. However, a substantial disadvantage is that such pyramid filters must be explicitly made (e.g., using 3D printing) per patient and treatment angle. Fabrication and QA of these filters would add to the treatment costs and patient treatment preparation time.

Although various experiments are ongoing, it is not clear if there is a different FE in irradiations in the plateau region compared to doses applied with the Bragg peak of the proton dose distribution. Most FLASH experiments with proton beams do not use the spread‐out Bragg peak, unless specific Bragg peak biology is investigated. In most experiments, one uses a single high energy, at which the volume of interest is in the plateau region before the Bragg peak. By reducing or removing degrader material crossed by cyclotron beams, a maximum intensity can be obtained. Usually, the beam energy is so high that the protons will cross the test material (cell culture or small animal) and stop behind it. This transmission technique is effective for preclinical experiments and is being used for an initial FLASH clinical trial.^25^ However, the proton transmission irradiations would give fairly similar dose distributions to X‐rays and reduce the tumor volume's dose conformity.

If static lateral beam shaping (non‐PBS) is desired, it can be easily accommodated by the classic DS system for larger fields, or single scattering for field diameters of approximately 1–2 cm.

In DS, the beam traverses a foil (or set of foils) made of a high Z material, for example, tungsten or lead. Due to the multiple scattering of the beam particles in the foil, the beam size increases after crossing these foils. A specially shaped foil then creates a flat beam profile at some distance behind the foil. Single scattering systems use an aperture to cut the center portion of the large, scattered Gaussian field, producing a tiny field of acceptable flatness, that is, 97% of the scattered field is cut, with the remaining 3% used as the flat field. Just in front of the patient, the lateral dimension of the beam is limited (for all scattering systems) by a collimator, which has the shape of the tumor, as seen from the beam direction.^93^ This collimator is patient‐ and gantry‐angle‐specific. Behind the scatter foil system, a range modulation wheel or a ridge filter is mounted to perform the distal spreading of the dose. The *DR*_ave_ will be in the order of a Gy/min, depending on the beam intensity and field size. When a modulation wheel is used, there will be a variation in dose rate over time over the irradiated volume.

Such modification of existing ion therapy systems may represent the easiest path to UHDR ion BDSs capable of inducing the FE. Indeed, with the current circular accelerators, the classical scatter technique (with a pyramid filter and a collimator, for clinical treatments of larger volumes) seems the only possible way to perform FLASH irradiations of deep‐lying tumors.

### 9.4 Scanning UHDR BDSs

Although passive BDSs may experience a short‐term renaissance, due to the practical need to provide an immediate solution, many users will not be ready to go back to the multiple drawbacks of using scattering BDSs. These include higher neutron dose to the patient, lower proximal dose conformity, the need to fabricate and test patient‐specific beam modifying devices, storage and disposal of activated parts/accessories, and more complicated treatment planning and commissioning. Hence, there is a need to develop a PBS FLASH system.

#### 9.4.1 UHDR PBS challenges

A beam with a typical diameter of 0.5 cm is scanned in the lateral plane in PBS. Up to the maximum field size, the used scan range is determined by the lateral dimensions of the tumor. Usually, after scanning an energy layer, the energy is varied, and another layer is scanned. Per energy plane, the scanning pattern is generally divided into voxels, each of which must be filled with a specific dose. This method is often referred to as “spot scanning.” Currently, in some facilities, “line scanning” is performed, in which the scanning speed and/or the beam intensity are varied with beam position. The advantages of both PBS methods, compared with DS, are better dose conformity, lower healthy tissue dose, and the convenience that no patient‐specific collimators and range limiting devices have to be made.

With PBS, the beam is at each location for a few milliseconds. At the most proximal locations, the total dose per field is applied within a few milliseconds. However, at more distal sites, the pencil beam passes multiple times for a few milliseconds to apply the Bragg peak at deeper located locations. This may cause dose rate variations in time in both the tumor volume and irradiated healthy tissue. Such variations will occur due to energy variations to spread the Bragg peak in‐depth, due to the lateral scanning of the pencil beam in the PBS technique. In PBS, due to both the lateral scanning and the energy changes, parts of the tumor volume are irradiated only once or a few times, during a few milliseconds, whereas other portions will be irradiated multiple times with millisecond pulses. The times between these irradiation pulses can vary between a few milliseconds and numerous seconds. The irregular dose rate distribution and PBS‐pulse interval distribution might distort the FE. Therefore, considering current PBS BDSs for UHDR applications, assuming sufficient accelerator flux, the primary challenge for FLASH deliveries is to achieve energy changes in a few milliseconds, and the second challenge is lateral dose delivery time.

#### 9.4.2 UHDR PBS developments

UHDR BDS are intimately linked with accelerator technology. Proton linacs, synchrotrons, and laser accelerators can modify the energy within the accelerator. Cyclotrons require an external energy degrading system. It is also possible to quickly degrade the irradiation volume energy in the BDS.^83^, ^94^, ^95^ However, it may be too challenging to achieve PBS UHDR from legacy accelerator types. Instead, the proton linac and laser accelerators intrinsically offer split‐second energy changes. Additional development is also needed in beam transport and monitoring (see next section). Furthermore, provided sufficient flux and fast energy changes, the lateral beam delivery speed will need to increase. Some developments for fast‐moving target irradiation might be used where ion scan speeds up to 120 m/s have been reported.^96^

**FIGURE 15:**
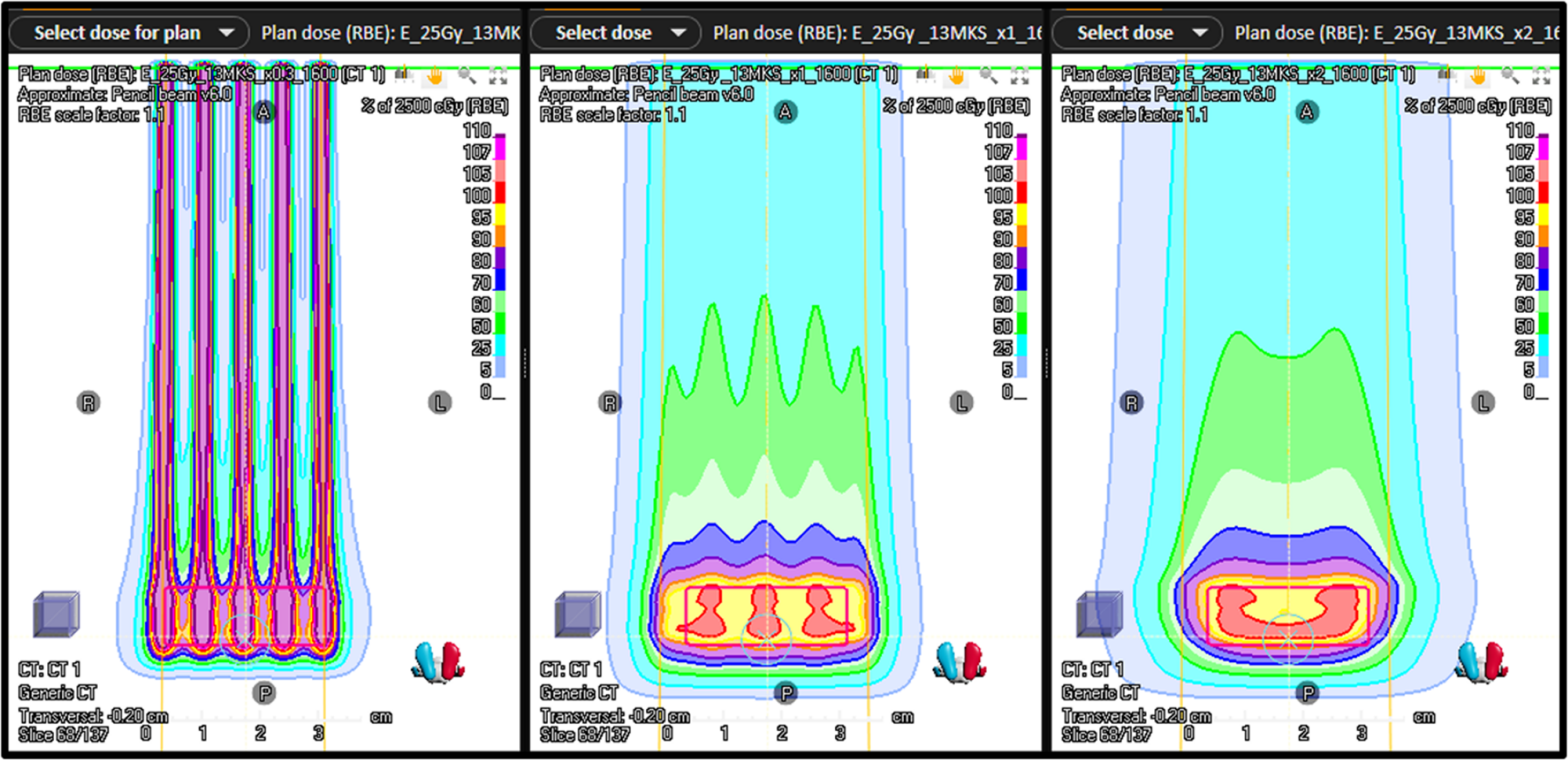
PBS treatment planning results (produced with RayStation, RaySearch AB, Stockholm) for a 6 cm^3^ target at 10 cm distal edge irradiated from (left to right) with 1, 3, and 6 mm *σ* in air at isocenter proton beam

The PBS spot size is a critical quantity for UHDR performance. Increasing the spot size reduces the total number of spots, boosting the local dose rate in the target, provided that the needed number of protons per spot is available. However, as shown in Figure 15, increased spot sizes may reduce the *DR*_ave_ in the proximal tissues where the FE is desired.^97^ Indeed, proton minibeams hold the promise of providing UHDR volumetric deliveries to limited volumes.^98^ Longitudinally, an increase of the energy spread in the proton beam would increase the width of the Bragg peak in‐depth so that fewer energy layers could be applied.

**FIGURE 16:**
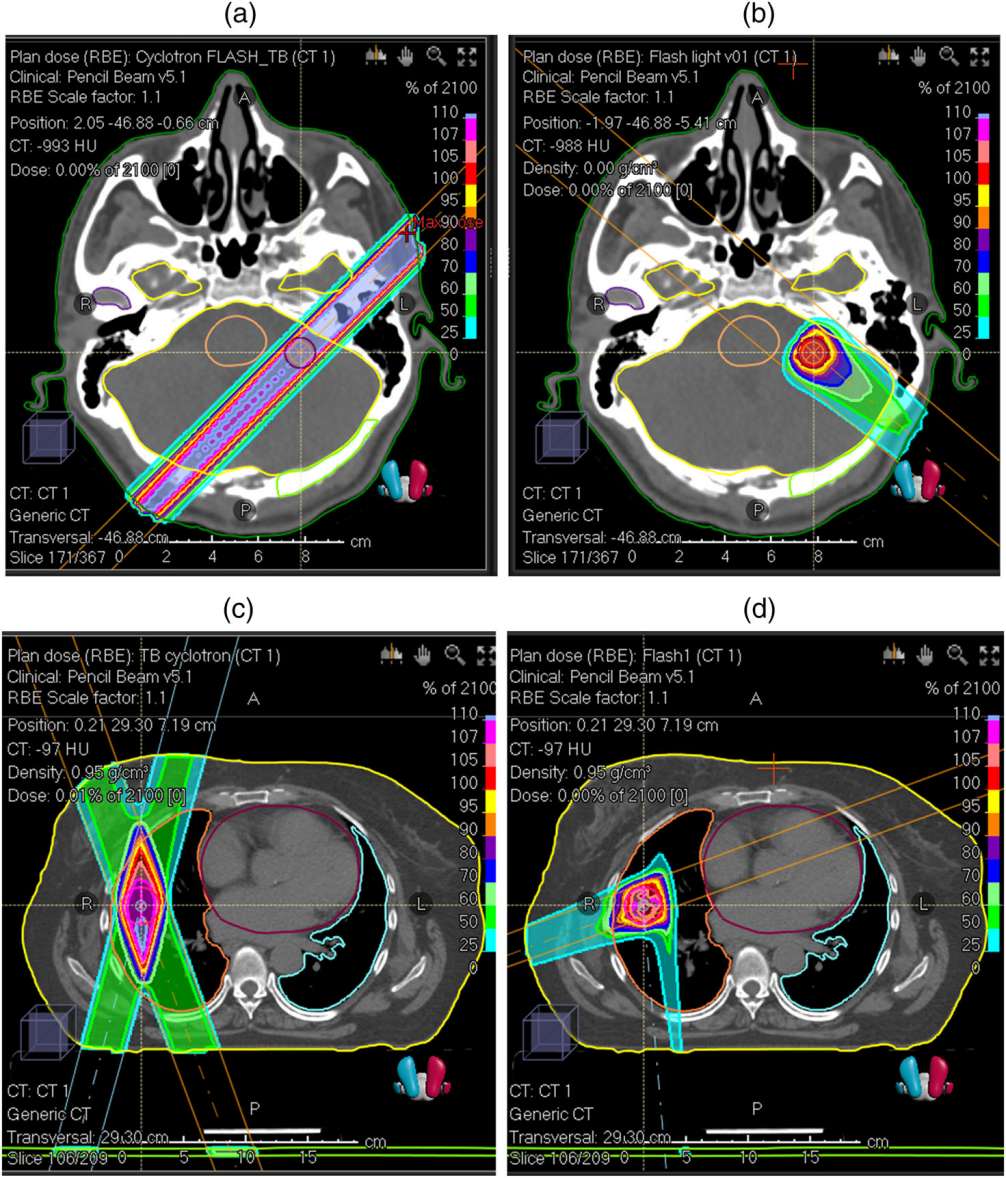
Comparison of PBS transmission (proton cyclotron) vs conformal (proton linac) 21 Gy single fraction UHDR plans for a 2 cm diameter brain and lung target (produced with RayStation, RaySearch AB, Stockholm). PBS conformal UHDR plans were not achievable without an additional beam modifying device (Sections 5.8 and 9.3) for the cyclotron model. The cyclotron UHDR transmission plans are shown for (a) brain and (c) lung. The linac UHDR transmission plans were similar, so they are not presented here. The linac UHDR conformal plans are shown in (b) brain and (d) lung. Comparatively, the calculated average dose rates (*DR*_ave_) are (a) 71, (b) 47, (c) 110, and (d) 44 Gy/s. Whereas the integral brain doses are 6.3 (a) 4.4 (b), and the integral lung doses are (c) 3.1 and (d) 1.9 Gy. Although the minimum and maximum UHDRs producing the FE are not currently clinically established, there was an observed tradeoff between dose rate and integral dose for the PBS transmission and conformal UHDR plans in this example. Although not reported quantitatively here, the Organ at Risk (OAR) doses are subjectively higher for the transmission plans than the conformal plans. This can be observed in the lung example, increased breast dose for the transmission plan (c) in comparison to zero breast dose for the conformal plan (d). In the brain example, the conformal plan (b) spares brainstem dose compared with the transmission plan (a)

Conformal PBS UHDR delivery may be feasible by combining fast energy layer changes with broad energy layer spacing. The concept and comparison to transmission PBS UHDR deliveries are presented for a brain and lung example in Figure 16. In the example, the cyclotron dose rate was determined by modeling existing published monoenergetic dose rates (Figure 12, orange curve) and assuming 1 s energy changes. The proton linac model used is represented in Figure 12, the yellow data series with 5 ms energy changes.

Comparing the PBS transmission and conformal UHDR plans, the conformal plans irradiate significantly less normal tissue and still achieve dose rates greater than 40 Gy/s. In contrast, the PBS UHDR transmission plans achievable maximum dose rates are higher. This is because PBS conformal UHDR deliveries scan over multiple energy layers even with fast energy changes, whereas the transmission deliveries only scan laterally once. The UHDR comparison between PBS transmission and conformal irradiations includes only physical dose. The DMF will have a significant effect in determining the ideal clinical application between the two techniques.

#### 9.4.3 UHDR delivery optimization

The dose rate delivery concepts discussed above are consistent with historic “forward treatment planning” where one “imagines” and then calculates the desired dose distribution and, in this case, dose rate distribution. Forward planning is a manual, iterative process that may or may not lead to adequate solutions for the requirements. Inverse treatment planning works in the opposite direction, where the result is taken as the input for an automated computer calculation. Arguably, given sufficient accelerator and BDS capability, inverse optimization of *DR*_ave_ and dose offers promise to understand and ideally utilize UHDR systems for producing the FE. Although current treatment planning systems (TPSs) cannot optimize for *DR*_ave_, such algorithms are currently under development.^99^

### 9.5 Laser proton dose delivery

**FIGURE 17:**
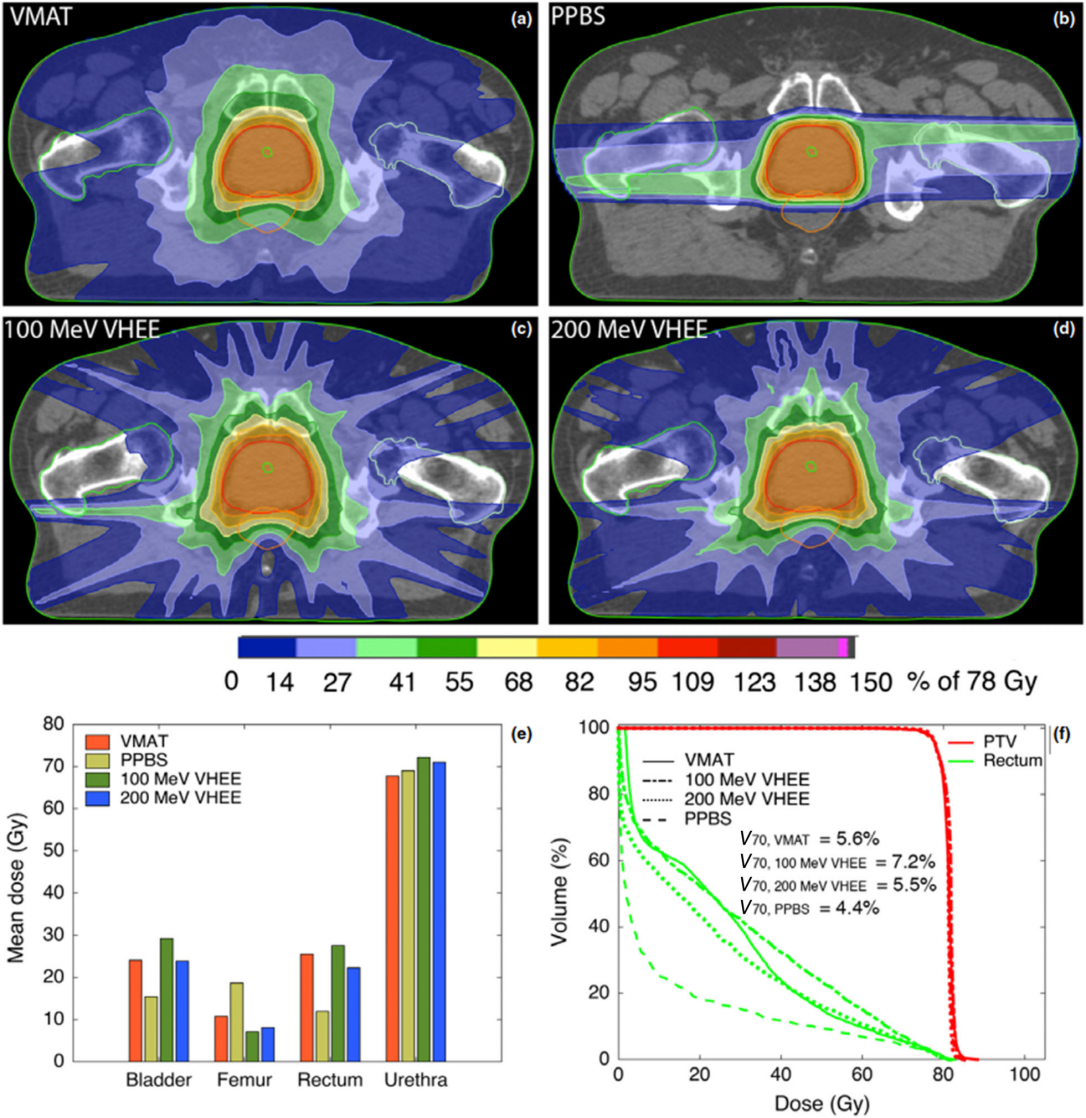
Comparison of treatment plans for prostate cancer. Treatment planning comparison between VMAT, PPBS, 100 MeV VHEE, and 200 MeV VHEE plans (a–d, respectively); (e) mean doses to the bladder, femurs, rectum, and urethra; (f) dose volume histogram for the planning target volume and rectum, together with the reported percent of prescription dose to 70% of the rectal volume (V70). Adapted with permission from Schüler et al.^102^

VHEE delivered by LPAs for cancer treatment was first considered in 2006, with focusing and nonfocusing beams.^100^ The calculated dose distribution in water, performed with experimental electron beam parameters, indicates a narrow radial profile and a longer penetration distance of VHEE than those of 20 MeV electrons, delivered by conventional accelerators. In 2009, dosimetry properties of laser‐accelerated VHEE beams were compared to a clinically approved 6 MV IMRT photon plan in a TPS study for prostate cancer.^101^ The study demonstrated a better target coverage of about 19%, favoring VHEE. In 2017, the subject was extended by comparing 6 MV volumetric modulated arc therapy (VMAT) photon to PBS, 100 MeV, and 200 MeV VHEE plans.^102^ Again, the VHEE plans were superior to VMAT plans (see Figure 17), with reduced mean organ at risk (OAR) dose and increased target conformity. Comparatively, the VHEE plan quality was usually intermediate between VMAT and PBS, for a variety of clinical cases, although VHEE was superior to PBS for specific shallow targets.^102^ Furthermore, the 3D dose distribution of VHEE produced by LPA, measured within a water phantom and compared with Geant4 simulations, confirmed the potential of VHEE for radiotherapy.^103^

## 10 HDR BEAM TRANSPORT AND MONITORING

This section will discuss how the beam transport between accelerator and patient should be adapted to enable protons to be used for experimental or clinical UHDR irradiations. Although we will confine the discussion to proton systems, most issues will also apply to treatments with ions.

### 10.1 UHDR beam transport

The proton beam from the accelerator is transported to the treatment room using a beam transport system. In most systems, including on gantries (Section 9.1), this covers a distance up to several tens of meters.

It is unclear whether interruptions of multiple seconds in the dose application will affect the FE. Such interruptions occur, for example, when the gantry rotates around the patient to another field. However, rotation of the gantry cannot be done faster, due to safety considerations. Developments include design of a BDS in which no mechanical motions are necessary to rotate the beam around the patient.^104^, ^105^

**FIGURE 18:**
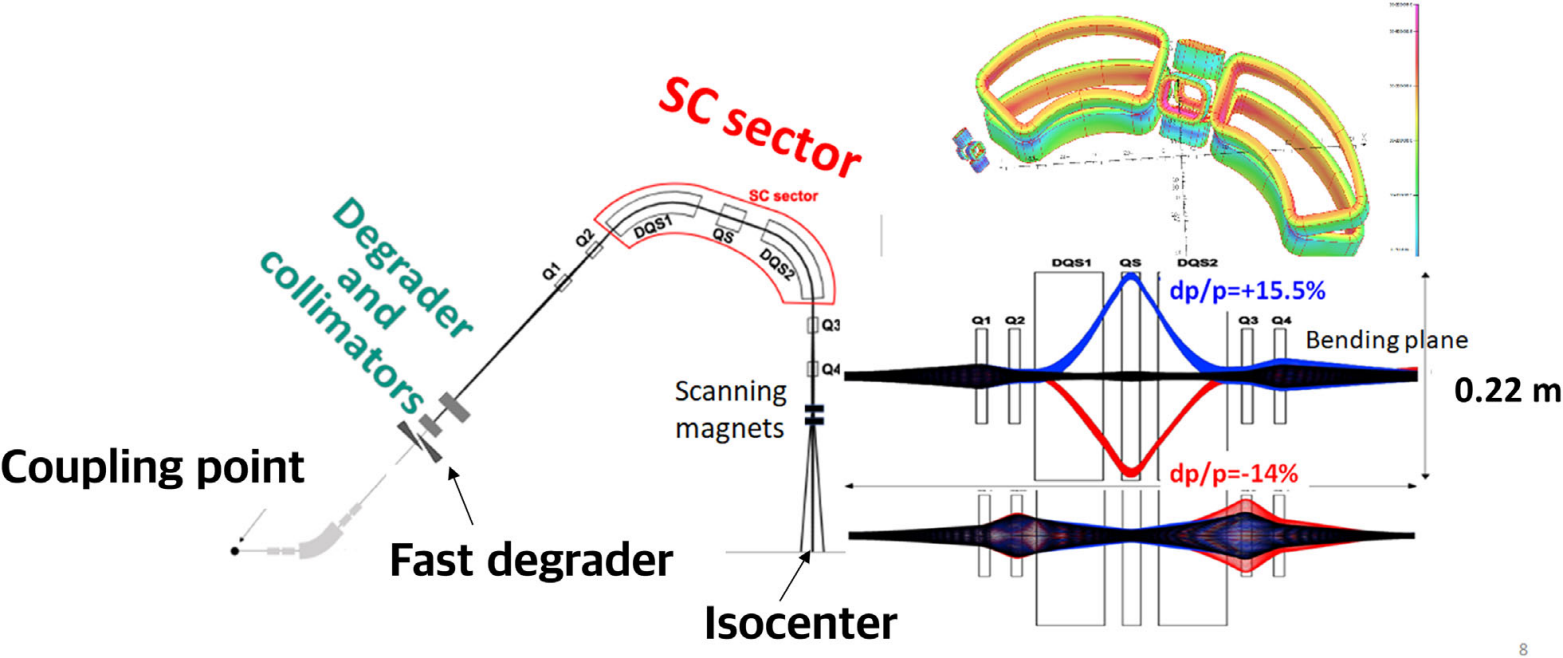
A gantry with a superconducting magnet system allows beam energies within a range of almost 30% so that these do not have to change their fields when beam energy changes after the degrader. Adapted with permission from Nesteruk et al.^107^

The beam transport magnets can be distinguished as quadrupole magnets to focus the beam and steering and bending magnets (dipole magnets) to aim the beam into specific directions. The magnet strengths are set automatically via the control system. However, since changing the magnetic fields takes some time (fraction of a second), this cannot often be done within a FLASH pulse. Up to some technical and financial limitations, the speed to change a magnet set can be increased. This can be done, for example, by more powerful magnet power supplies, other magnet coil designs, and by thin lamination of the magnets. SC magnet systems, which have a large energy acceptance (“achromatic”), are being designed. Their fields need to be changed only once or even when another beam energy is going through.^106^, ^107^ An example of such a gantry,^107^ which includes a fast degrader, is shown in Figure 18.

**FIGURE 19:**
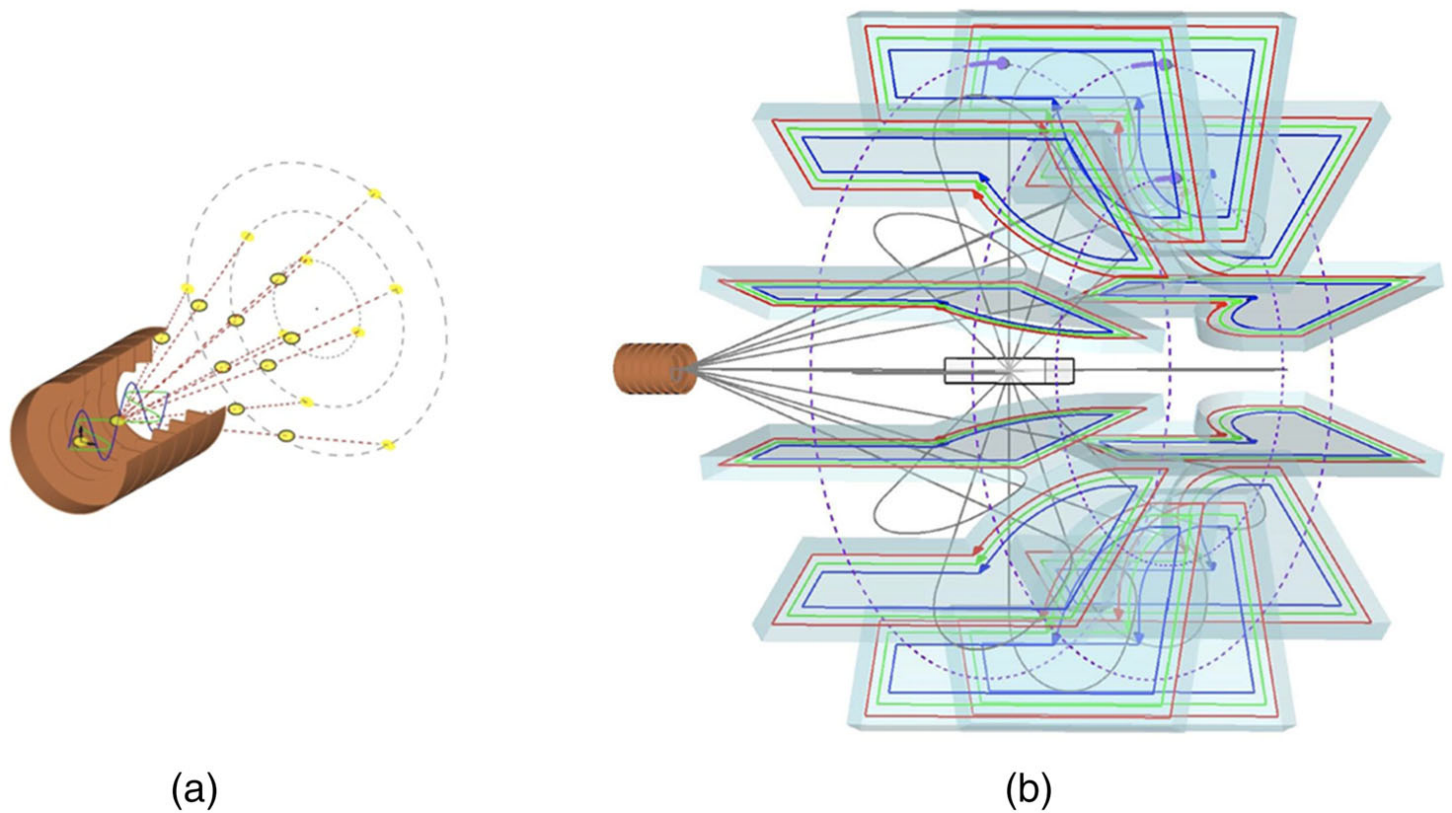
Schematic illustration of (a) An RF solid angle deflector with variable polarization, feeding into (b) static superconducting coils covering 12 different angles. Adapted with permission from Fang et al.^65^

Considering motionless gantries, the aim is to provide continuous^104^, ^105^ or sufficient fixed angles for conformal target irradiation. Recently, a fixed design using 12 SC magnets (see Figure 19) was proposed for UHDR delivery.^65^ The FLASH gantry concept aims to provide ultra‐fast layer scanning of 10 µs or less using a single pulse from a FLASH linac.^65^

### 10.2 UHDR beam and dose monitoring

In beam transport systems and the nozzle, the demands on beam diagnostics and beam monitoring will be more complicated, since it must have sensitivity over an extensive intensity range (nA–µA) and react very quickly to enable a beam interruption (interlock), if required. Hence, beam diagnostics, dose monitors, and pencil beam position measurements must be adapted, or recalibrated, for the high intensity of the FLASH conditions. If intended to excite an interlock, in case of failure detection, electronics and beam switch‐off systems must work at a sufficiently high speed to be effective.

Because it must be active during treatment, it would be advantageous if beam diagnostics would not intercept the beam. In most beam transport systems and nozzles, beam diagnostics and dose monitors are based on ionization chambers with one (for intensity measurement) or multiple (for beam profile and beam position) electrodes. In UHDR conditions, these chambers may suffer from saturation effects, dependent on beam intensity and beam size. Beam stoppers provided with a Faraday cup mounting are used for accurate beam intensity measurements over the entire intensity range. However, since they intercept the beam, these can only be used offline. Therefore, new ways to operate existing devices, or new beam diagnostics and dosimetry monitors, must be used when UHDR irradiations are performed. Developments are in progress and, as an example, the recently developed dielectric‐filled reentrant cavities will be discussed.

#### 10.2.1 A novel beam current monitor for UHDR

**FIGURE 20:**
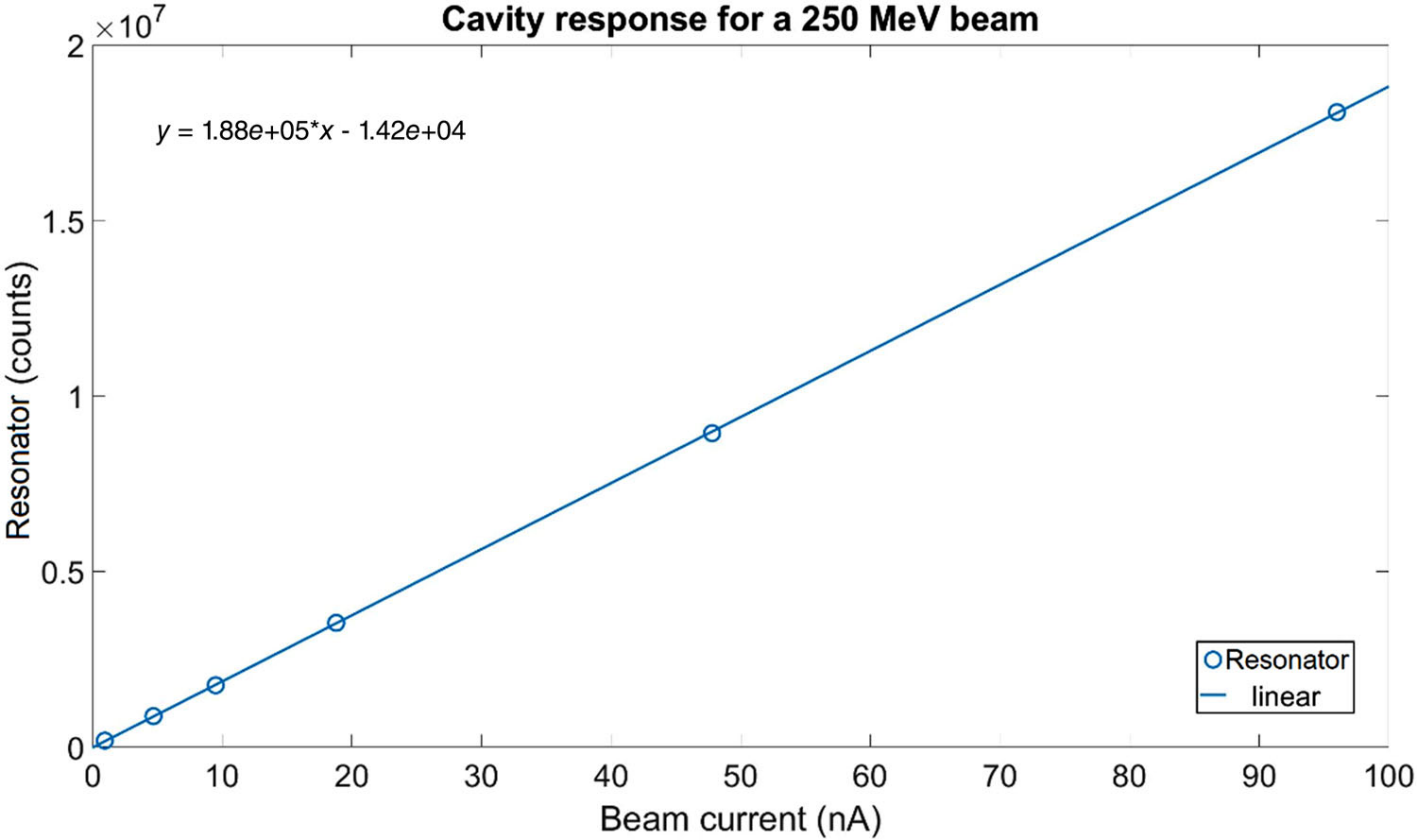
Signal detected in the resonator as a function of the intensity (nA) of a 250 MeV proton beam

The principle to apply such a cavity resonator as a beam current monitor (BCM) is described.^108^ The BCM design follows the excitation of an electromagnetic resonance in an LC resonator (inductor L, and a capacitor C, combined in series to act as an electrical resonator) mounted around the proton beam. This resonance is excited by the, for example, ∼70 MHz pulses in the beam from a cyclotron. An antenna detects the resonance in the cavity. By partly filling the cavity with a dielectric material, the cavity dimensions can be limited, and a maximum pickup coupling coefficient is achieved between the proton beam and the antenna. Although initially designed to detect low beam intensities (0.1–10 nA), the BCM's response remains linear at high intensities, as shown in Figure 20.

**FIGURE 21:**
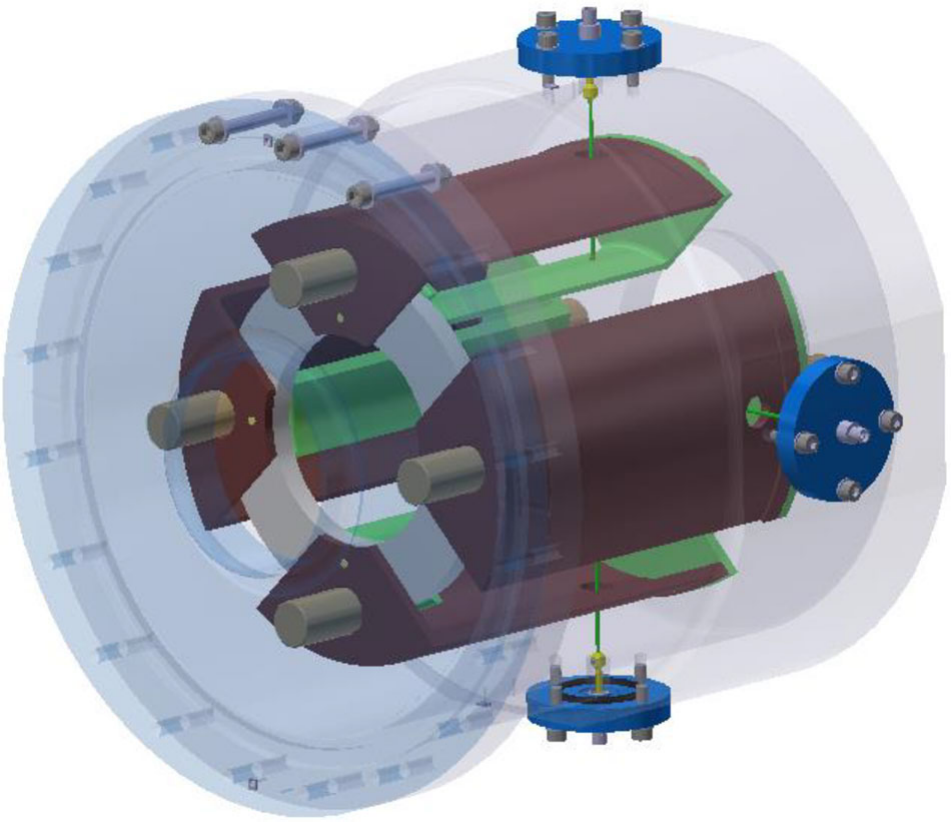
A noninterceptive beam position monitor based on four resonating cavities surrounding the proton beam

Based on the same principle, a beam position monitor has also been developed.^108^, ^109^ In this monitor, the beam is surrounded by four separate cavities, as shown in Figure 21. The amplitude of the resonance excited in a cavity is proportional to the offset of the beam from the central axis into the direction of the cavity.

Similar to the BCM, this beam position monitor also measures in a purely noninvasive manner. Thus, both types of monitors have the advantages of relatively compact size and a strong coupling to the beam, enabling them to work in an intensity range from a fraction of a nA to at least intensities needed for FLASH irradiations.

#### 10.2.2 UHDR dose monitoring

Conventional TICs used in clinical practice experience significant charge recombination when exposed to pulsed UHDR beams. This results in a saturation of the measured signal. The applied correction factors rapidly increase with *D*_pulse_, already above 1.5 for 2 µs pulses carrying 1 Gy and becoming higher than 3 when the pulse dose surpasses 10 Gy.^110^ Such high correction factors are well above the maximum values recommended by the International Atomic Energy Agency (IAEA) International Code of Practice for dosimetry of external electron beams (IAEA TRS‐398^111^) and make the dose estimation inaccurate. The lack of an appropriate dose monitoring device presents a severe problem for the clinical implementation of pulsed UHDR beams. The use of the AC Current Transformers (ACCT) as online beam monitors was recently validated, first on a preclinical Oriatron eRT6 electron irradiator and, later, on a Mobetron device, the first commissioned clinical UHDR electron linac.^51^ An ACCT is a toroid sensor composed of a conductive winding that measures the induced current of the charged particle beam passing through it. Placement of the ACCT immediately outside the beam exit window does not interfere with the beam, as long as the inner radius of the winding is larger than the beam itself.

Regarding live monitoring of charged particle beams, an ACCT offers advantages over TICs in several aspects. An ACCT provides a cumulative beam output charge and a temporal shape of the beam pulses with a sub‐nanosecond time resolution. Time‐resolved waveforms representing the pulse current can be further analyzed to extract data such as the integrated pulse charge or the pulse duration time for each pulse. On the other hand, an ACCT does not provide information about the beam's flatness and symmetry, which is the case with modern transmission chambers. However, the absence of the saturation effect at high doses per pulse makes ACCTs suitable for live dose monitoring in the case of pulsed FLASH beams. A relationship can be established between the ACCT signal and the absorbed dose, similar to how it is usually done for the transmission chamber readout.

Although ACCT technology is attractive for UHDR monitoring, further developments in TIC technology are also occurring. TICs have been standard in radiotherapy devices since the 1950s and offer the advantage of minimal (usually less than 1% total) adjustment for accurate radiation beam dosimetry, including for ion beams.^112^ The high instantaneous charge density of UHDR should require a high electric field strength in the TIC plate gap. Higher electric field strengths can be produced by reducing the plate gap, increasing the bias, and so on. Alternative filling gasses can also be beneficial in limiting recombination. Researchers at the Physikalisch‐Technische Bundesanstalt, in Germany, determined that TIC corrections might be held within 3% for certain UHDR electron beams up to 3 Gy D_pulse_.^113^ Such developments are required for the use of UHDR in clinical applications.

## 11 CONCLUSIONS

Only a few beams have reproduced the FE in vivo. Data from electron‐based experiments indicate that *t*/*DR*_ave_ and *D*_pulse_/*DR*_pulse_ are likely critical parameters for the FE. Also, irradiation times shorter than 0.5 s (DR_ave_ greater than several tens of Gy/s) and pulse doses greater than 0.2 Gy are required to achieve the FE.

Because all proton FLASH results have been obtained with quasi‐continuous beams devoid of beam macrostructure, *DR*_ave_ is considered as a significant parameter for proton beams. Proton *DR*_ave_ values greater than 80 Gy/s are observed to elicit the FE. PBS delivery has also been shown to produce the FE, but the role of scanning speed and spot size on the FE needs further investigation. Although it is not yet clear if, and under what conditions, lateral and longitudinal dose spreading within the required short FLASH pulse are needed. In principle, the characteristics of beams from the isochronous cyclotron and the synchrotron seem to be sufficient to perform transmission FLASH treatments in restricted clinical situations. In addition, limited clinical proton UHDR treatments are being performed.^25^ The proton UHDR deliveries are transmitted through the patient. Therefore, in the authors’ opinion, because proton transmission UHDR deliveries do not take advantage of the stopping property of protons, further development is required to produce UHDR proton systems capable of conformal dose delivery, enabling conformal PBS FLASH treatments.

Further UHDR technological development is needed to obtain higher dose rates. It should be noted, however, that for making decisions on new accelerators, major instrumentation, and dose delivery methods, there is a strong need for answers to at least the following questions:

1. Experimental results are needed to obtain quantitative specifications on ranges for the dose, dose rates, volume, and time dependence of dose delivery.
2. Do local dose rate variations within the target or within irradiated healthy tissue play a role for observing the FE?
3. To obtain an acceptable volumetric FE, can sub‐volumes of tissues be irradiated sequentially, and does each sub‐volume require irradiation within the FE conditions?
4. Could dose conformity be compromised when the high dose rates sufficiently reduce complication probability due to the FE?
5. What role do time intervals play in the dose delivery process for observing the FE? For example, how are the time intervals between pencil beams, different energies, and different gantry angles related to FE observations?

Despite these scientific and medical unknowns, the authors have attempted to identify potential key technologies that may enable UHDR to provide FLASH deliveries in‐patient. These need to be focused on how to combine the longitudinal dose spreading (i.e., energy variations) with the lateral dose spreading (e.g., by pencil beams), within the FLASH conditions. Considering the accelerators in detail, cyclotrons and synchrocyclotrons are notably limited in machine output for beam energies below their maximum extraction energy, due to the need for energy degraders. In addition to absorbing beam energy, degraders also reduce the beam current because of proton loss. Therefore, cyclotrons and synchrocyclotrons may require placing the energy degrader proximal to the patient, but this may be unattractive, due to neutron production and concomitant additional whole‐body dose. Synchrotron output current does not suffer output energy dependence to the same degree as the other circular accelerator types. Because the maximum number of particles with a filling and acceleration cycle of the synchrotron is limited, this may need an undesired macrostructure of their proton delivery, sending “proton spills” every 5–7 s. Although the proton beam spills can be instantaneously UHDR, the synchrotron applicability to observing the FE will be subject to the dependency on this macro beam structure and is still under investigation.

Linear proton accelerators show more promise for conformal proton UHDR deliveries. Specifically, the proton linac beam current does not depend on the selected energy, and the energies can be changed within 5 ms. Hence, provided enough nominal proton flux and sufficient scanning speed, some conformal UHDR fields may be produced.^97^ Also, proton linacs may offer attractive *D*_pulse_ and *DR*_pulse_ quantities to achieve the FE as with electron linacs. Laser accelerators also show promise for proton UHDR, either as full energy accelerators or high‐output, stronger energy proton sources (i.e., as a linac injector), reducing the space‐charge limitation. Transmission electron UHDR can also be considered using VHEE, most likely employing linacs or laser accelerators. Although much progress has been made with laser accelerators, they remain in the preclinical setting, and significant further development would be required to produce a clinical system. These observations are summarized in Table 7.

**TABLE 7:** Predicted development path for clinical UHDR systems

	Electron	Proton	Heavy Ions
Existing UHDR accelerators	Linac	Cyclotron, synchrotron, synchrocyclotron	Synchrotron
Existing UHDR beam delivery	Low energy, small field size	Only maximum energy, field size up to 10 cm	Variable, small field size
Existing clinical use	Superficial, IORT (soon)	Transmission, body extremities, small fields	None
Unmet clinical need	Conformal, deep‐seated targets, larger field sizes	Conformal beam stopping in target, deep‐seated targets, larger field sizes	Conformal beam stopping in target, deep‐seated targets, larger field sizes
Enabling accelerator technology	High energy linac, laser	High energy linac, laser	High‐flux synchrotron, linac
Enabling beam delivery technology	VHEE, UHDR dose monitoring	Ultra‐fast energy changes, rapid lateral scanning, UHDR monitoring	Ultra‐fast energy changes, fast lateral scanning, UHDR monitoring

## CONFLICT OF INTEREST

Jonathan Farr holds a senior management position at ADAM SA, Meyrin, Switzerland and is a shareholder in Advanced Oncotherapy, plc, London, UK.
